# Supplementary material for: Investigation of Chemical Composition and Biological Activities of Ajuga pyramidalis—Isolation of Iridoids and Phenylethanoid Glycosides
Source: Metabolites. 2023 Jan 14;13(1):128. doi: 10.3390/metabo13010128 (PMC9860644; doi:10.3390/metabo13010128)
Supplement: Supplementary file 1 [file metabolites-13-00128-s001.zip › metabolites-2146721-supplementary.pdf]

Supporting Information

Investigation of Chemical Compositions and Biological Activities of *Ajuga pyramidalis* – Isolation of Iridoids and Phenylethanoid Glycosides

Anthonin Gori [1,2], Benjamin Boucherle [1], Aurélien Rey [2], Maxime Rome [3], Caroline Barette [4], Emmanuelle Soleilhac [4], Christian Philouze [5], Marie-Odile Fauvarque [4], Nicola Fuzzati [2] and Marine Peuchmaur [1,*]

[1] Univ. Grenoble Alpes, CNRS, DPM, 38000 Grenoble, France
[2] CHANEL Parfums Beauté, 93500 Pantin, France
[3] Jardin du Lautaret, CNRS, Université Grenoble Alpes, Grenoble, France
[4] Univ. Grenoble Alpes, CEA, Inserm, IRIG, BGE, F-38000 Grenoble, France
[5] Univ. Grenoble Alpes, CNRS, DCM, 38000 Grenoble, France
[*] Correspondence: marine.peuchmaur@univ-grenoble-alpes.fr; Tel.: +33476635295

List of supplementary files:

Figure S1. Global composition of the extracts of *A. pyramidalis*

Figure S2. ^{1}H NMR spectrum of echinacoside (CD_3OD, 500 MHz)

Figure S3. ^{13}C NMR spectrum of echinacoside (CD_3OD, 125 MHz)

Figure S4. COSY spectrum of echinacoside (CD_3OD)

Figure S5. HSQC spectrum of echinacoside (CD_3OD)

Figure S6. ^{1}H NMR spectrum of verbascoside (CD_3OD, 500 MHz)

Figure S7. ^{13}C NMR spectrum of verbascoside (CD_3OD, 125 MHz)

Figure S8. COSY spectrum of verbascoside (CD_3OD)

Figure S9. HSQC spectrum of verbascoside (CD_3OD)

Figure S10. HMBC spectrum of verbascoside (CD_3OD)

Figure S11. ^{1}H NMR spectrum of teupolioside (CD_3OD, 500 MHz)

Figure S12. ^{13}C NMR spectrum of teupolioside (CD_3OD, 125 MHz)

Figure S13. COSY spectrum of teupolioside (CD_3OD)

Figure S14. HSQC spectrum of teupolioside (CD_3OD)

Figure S15. HMBC spectrum of teupolioside (CD_3OD)

Figure S16. ^{1}H NMR spectrum of harpagide (CD_3OD, 400 MHz)

Figure S17. ^{13}C NMR spectrum of harpagide (CD_3OD, 100 MHz)

Figure S18. COSY spectrum of harpagide (CD_3OD)

Figure S19. HSQC spectrum of harpagide (CD_3OD)

Figure S20. HMBC spectrum of harpagide (CD_3OD)

Figure S21. ^{1}H NMR spectrum of 8-*O*-acetylharpagide (DMSO-d_6, 400 MHz)

Figure S22. ^{13}C NMR spectrum of 8-*O*-acetylharpagide (DMSO-d_6, 100 MHz)

Figure S23. COSY spectrum of 8-*O*-acetylharpagide (DMSO-d_6)

Figure S24. HSQC spectrum of 8-*O*-acetylharpagide (DMSO-d_6)

Figure S25. HMBC spectrum of 8-*O*-acetylharpagide (DMSO-d_6)

Figure S26. Acetylation reaction: HPLC chromatograms

Figure S27. Expression analysis of selected RNA for keratinocytes (NHEK) from donor 1

Figure S28. Expression analysis of selected RNA for keratinocytes (NHEK) from donor 2

Figure S29. Expression analysis of selected RNA for keratinocytes (NHEK) from donor 3

Figure S30. Interannual variability study on *Ajuga pyramidalis* ethanolic extracts

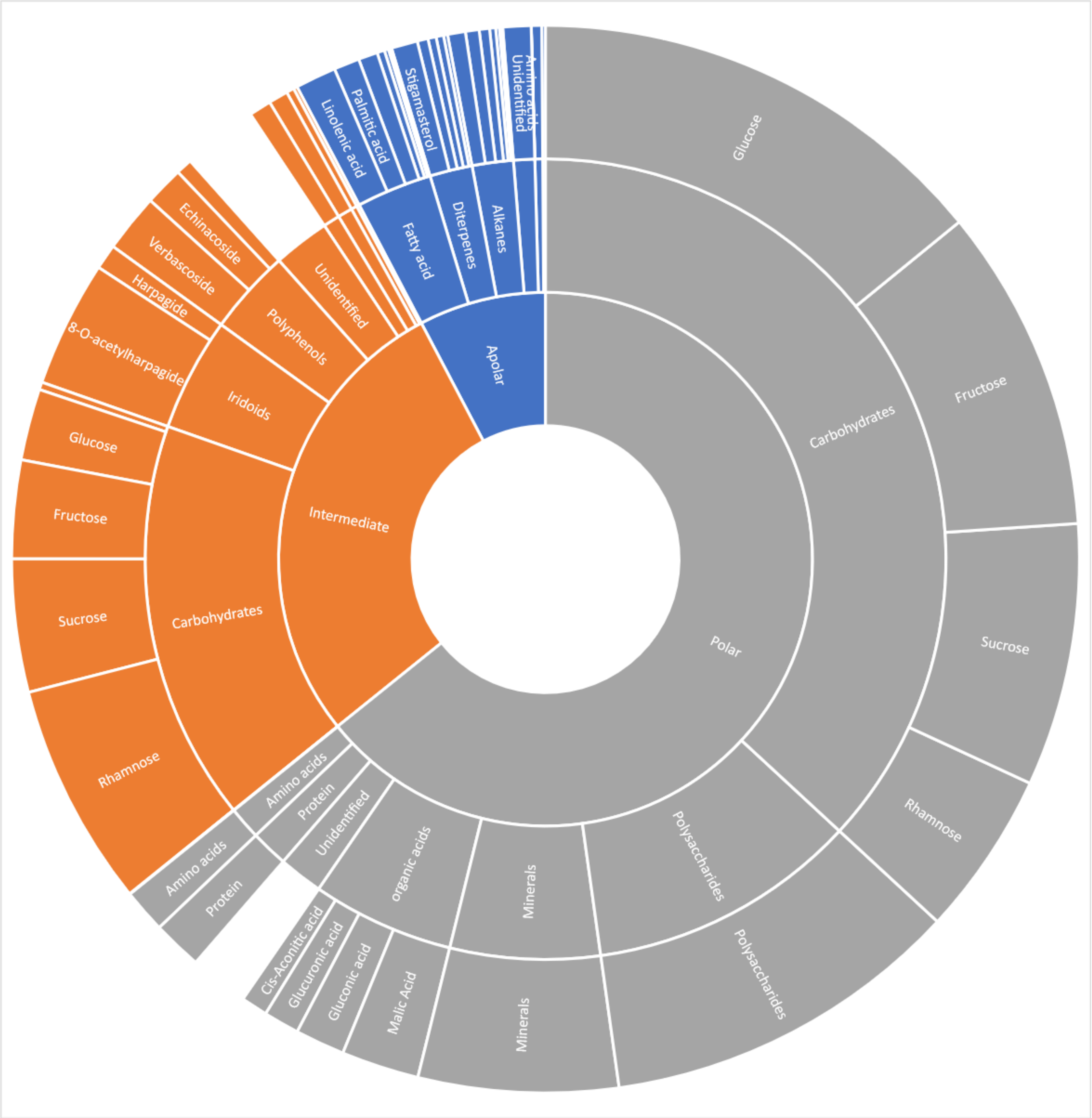

Figure S1. Global composition of the extracts of *A. pyramidalis* (in blue: the apolar extract, in orange: the intermediate extract and in grey: the polar extract).

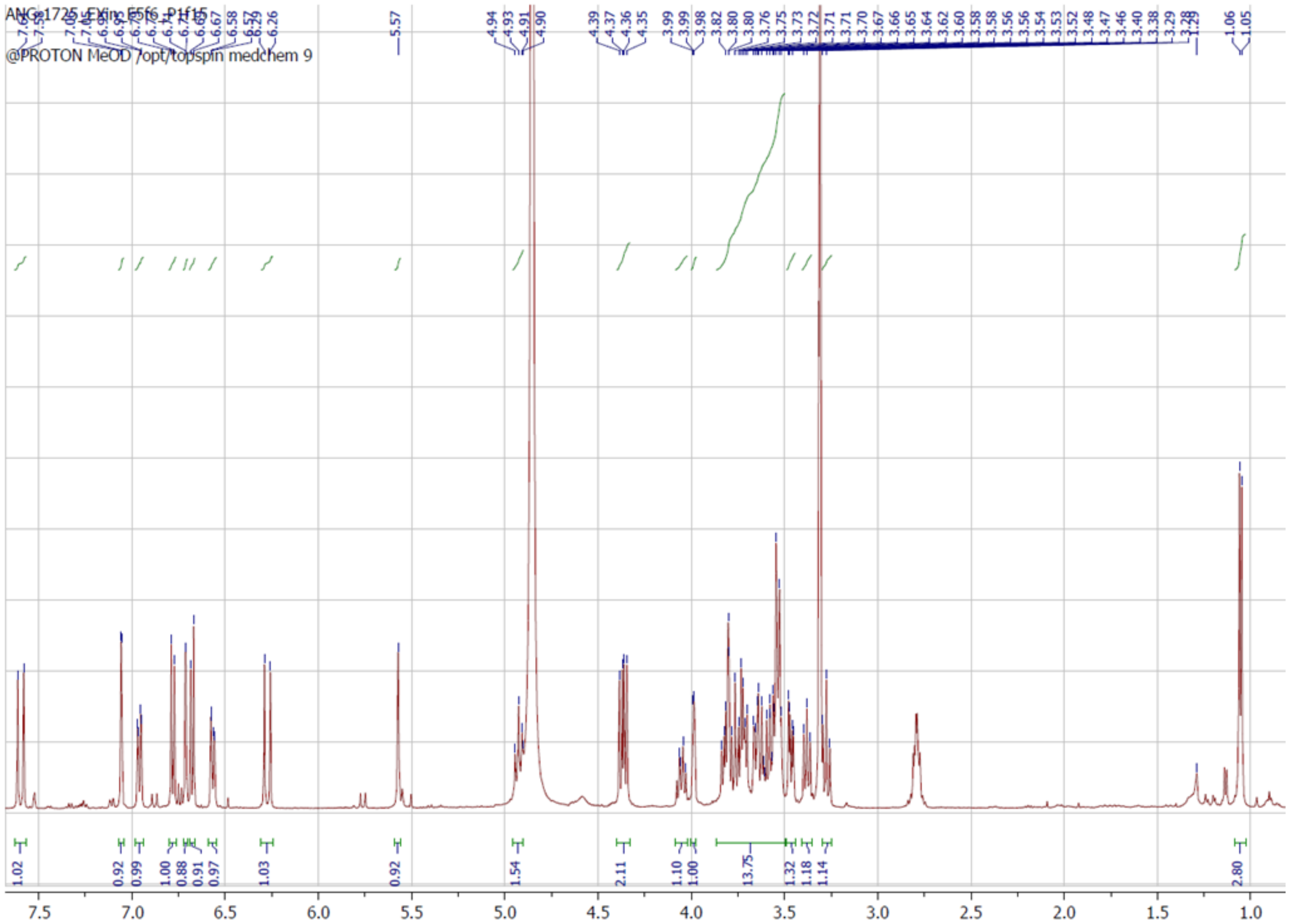

Figure S2. ^{1}H NMR spectrum of echinacoside (CD_3OD, 500 MHz)

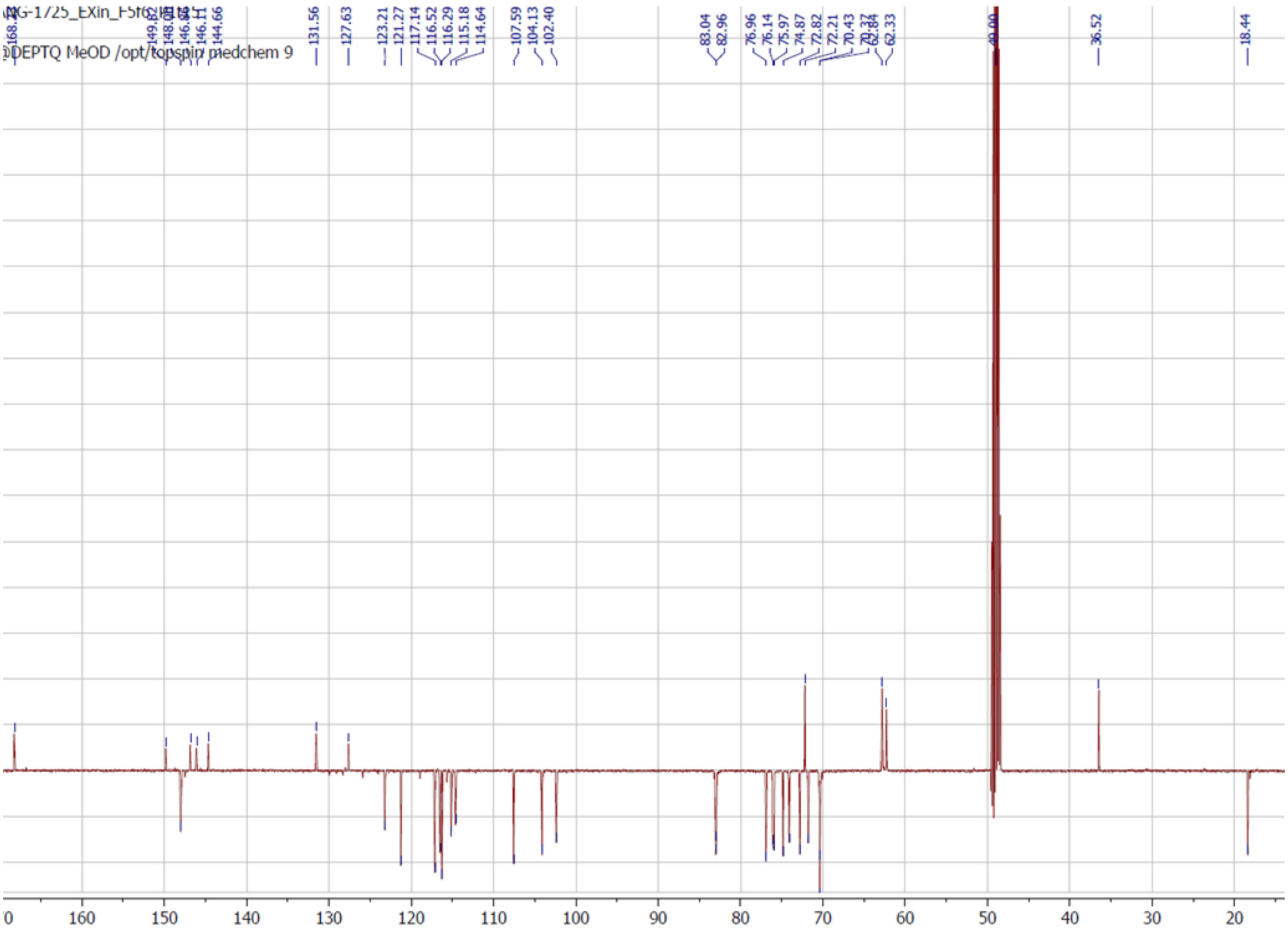

Figure S3. ^{13}C NMR spectrum of echinacoside (CD_3OD, 125 MHz)

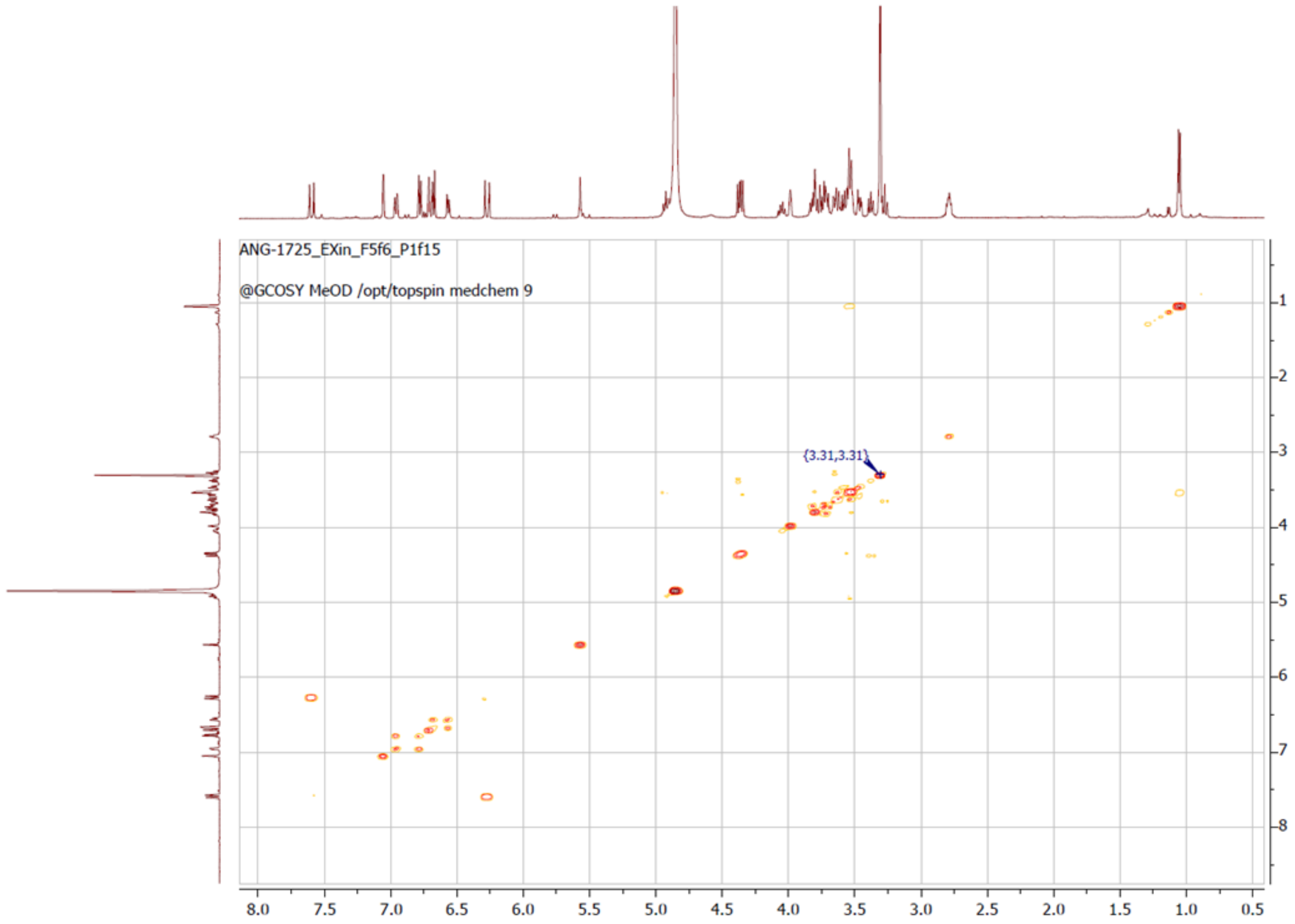

Figure S4. COSY spectrum of echinacoside (CD_3OD)

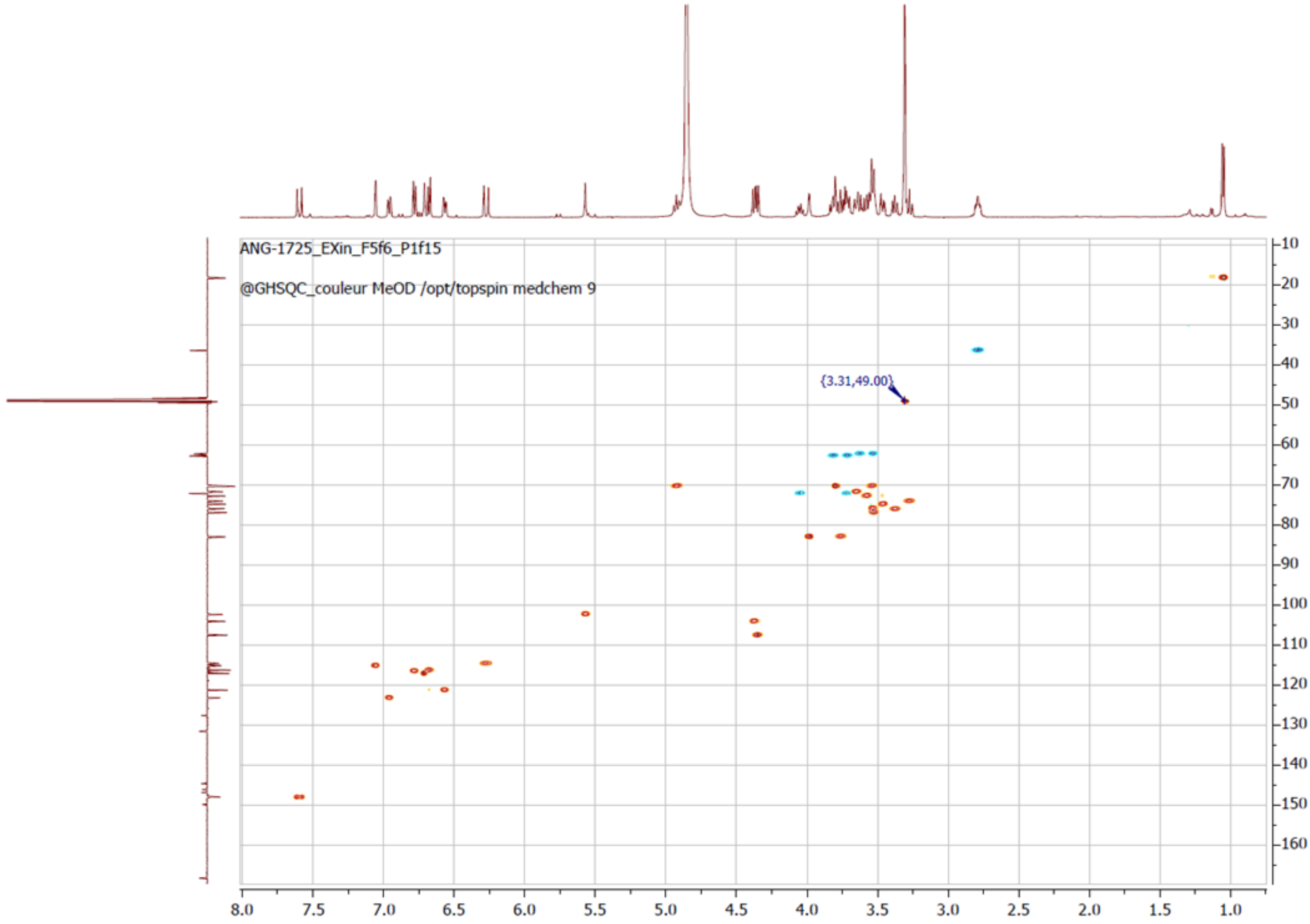

Figure S5. HSQC spectrum of echinacoside (CD_3OD)

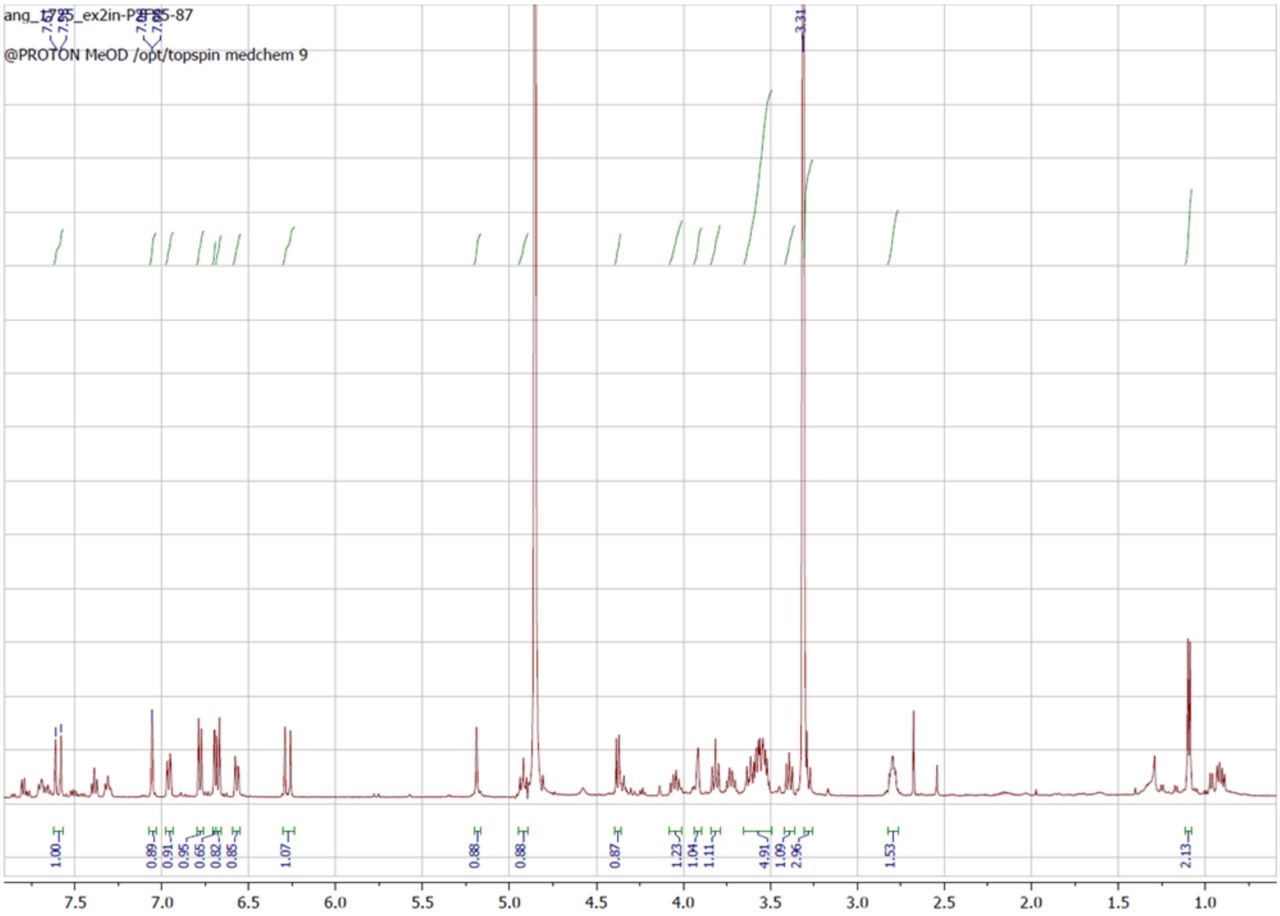

Figure S6. ^{1}H NMR spectrum of verbascoside (CD_3OD, 500 MHz)

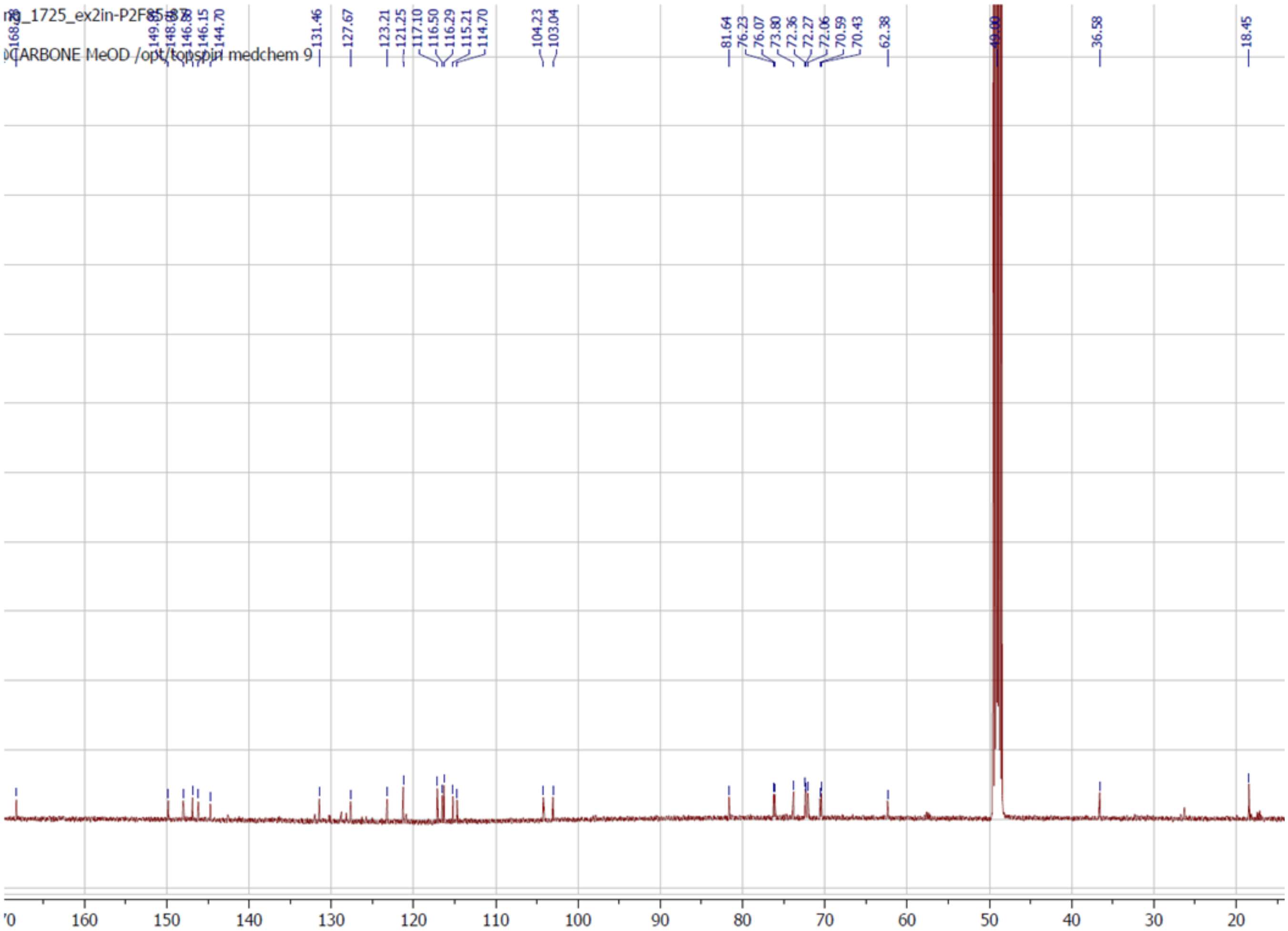

Figure S7. ^{13}C NMR spectrum of verbascoside (CD_3OD, 125 MHz)

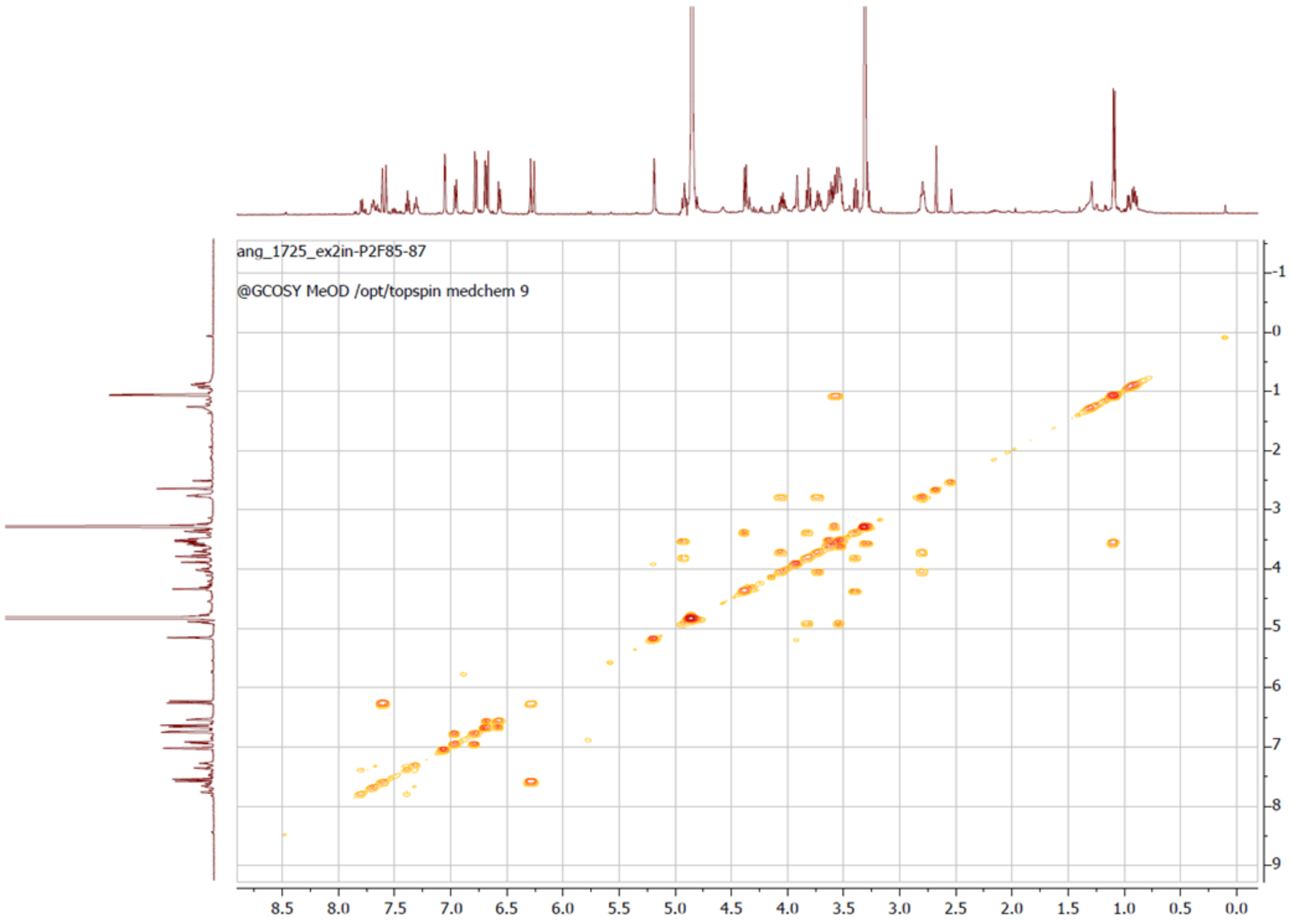

Figure S8. COSY spectrum of verbascoside (CD_3OD)

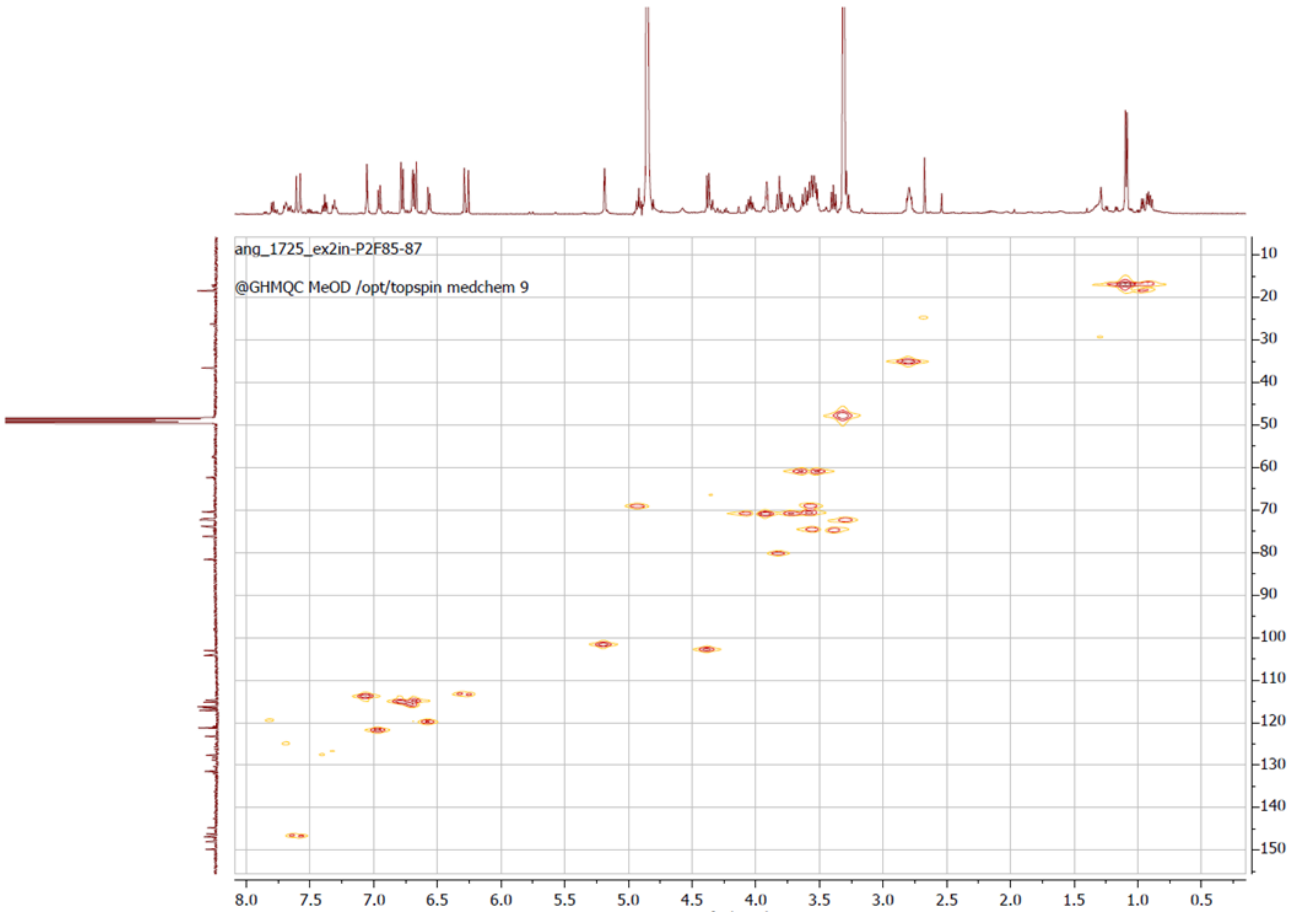

Figure S9. HSQC spectrum of verbascoside (CD_3OD)

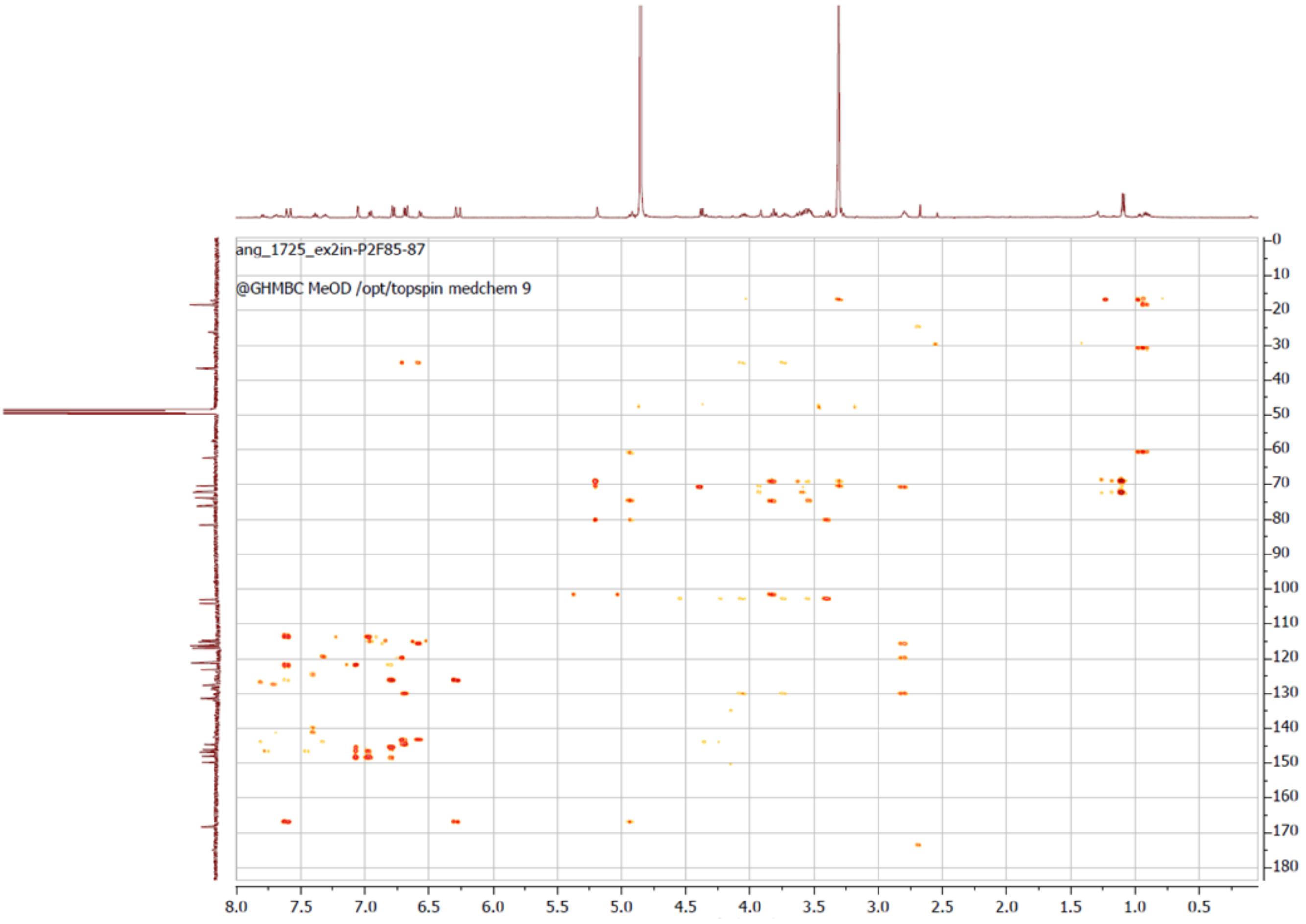

Figure S10. HMBC spectrum of verbascoside (CD_3OD)

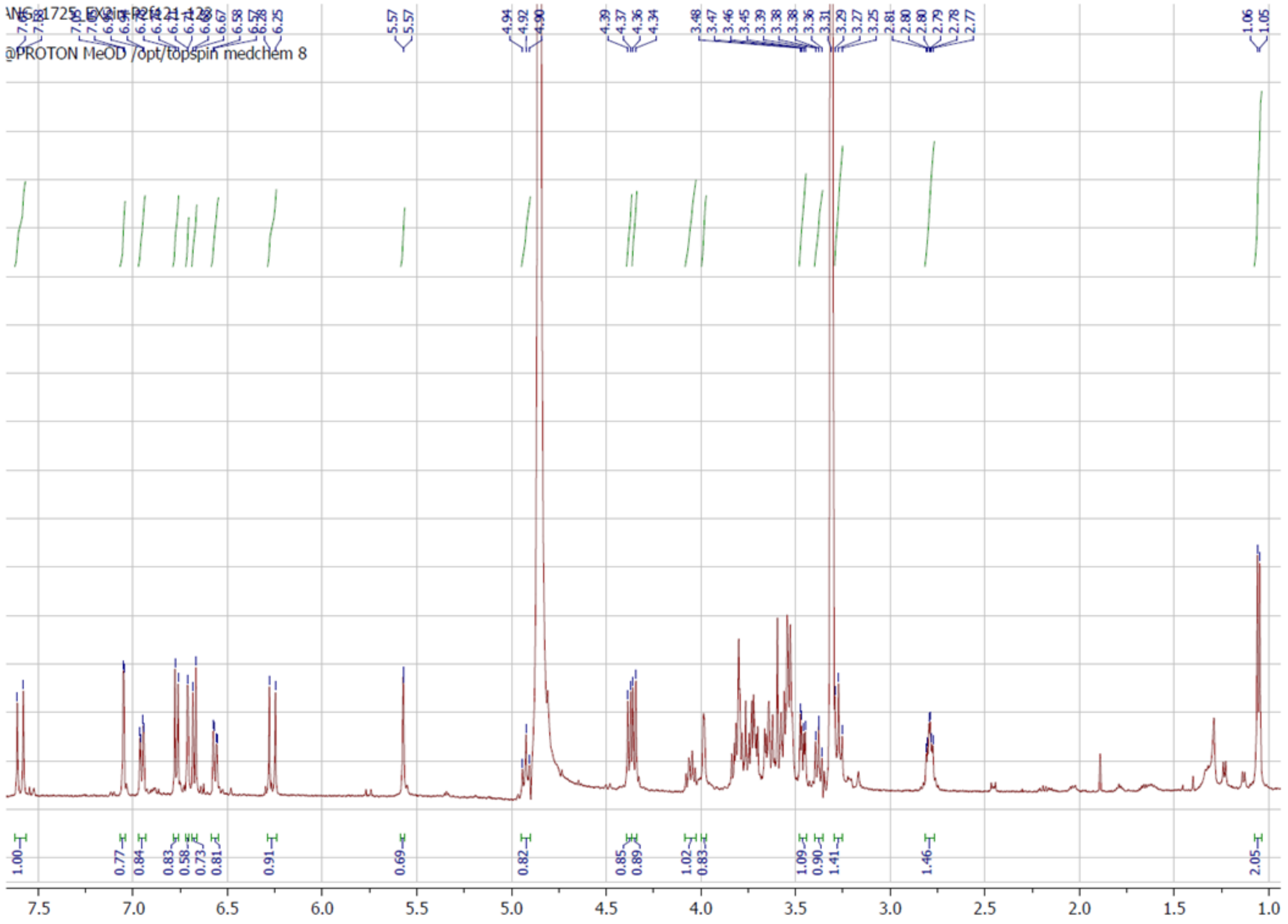

Figure S11. ^{1}H NMR spectrum of teupolioside (CD_3OD, 500 MHz)

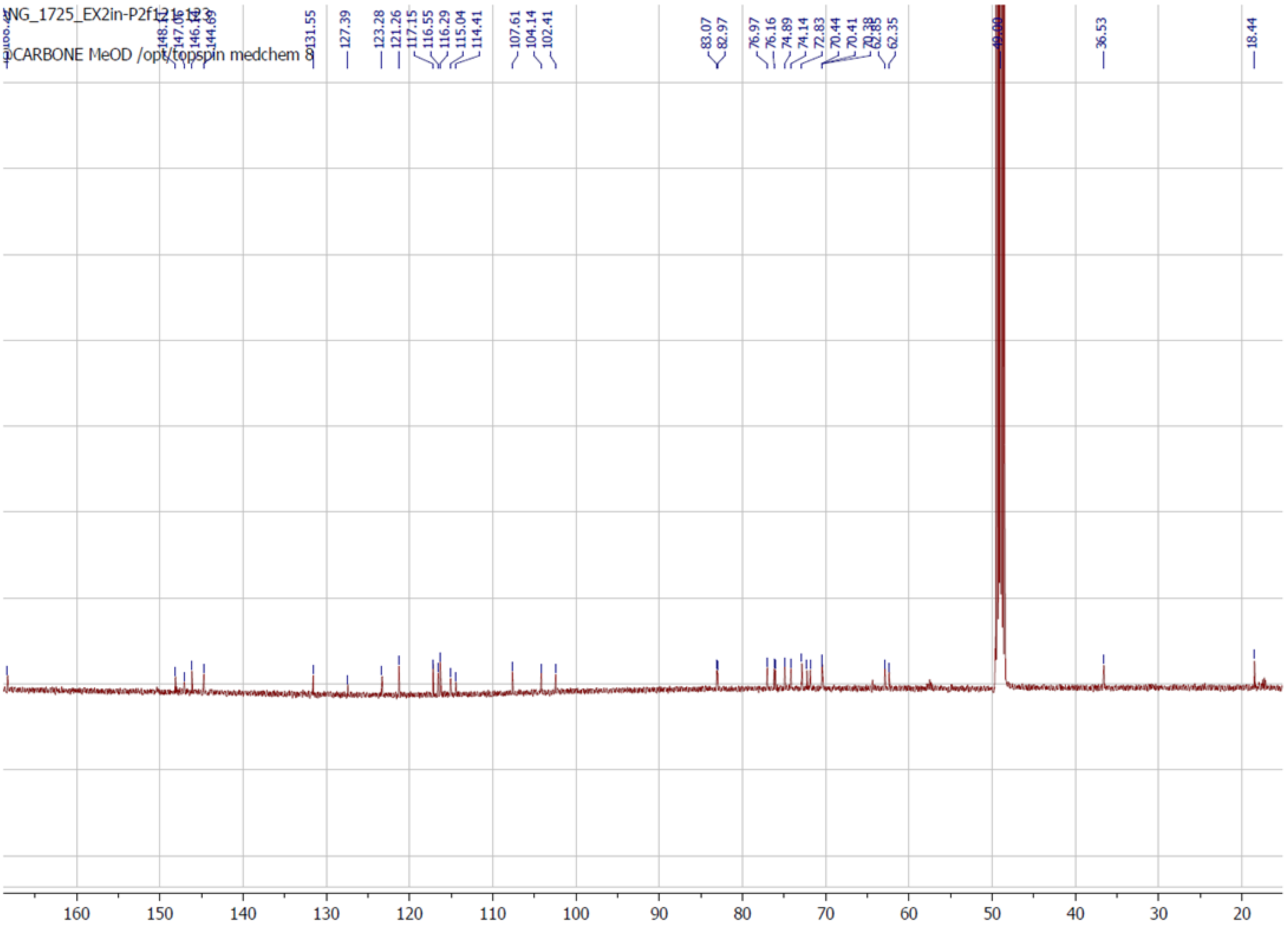

Figure S12. ^{13}C NMR spectrum of teupolioside (CD_3OD, 125 MHz)

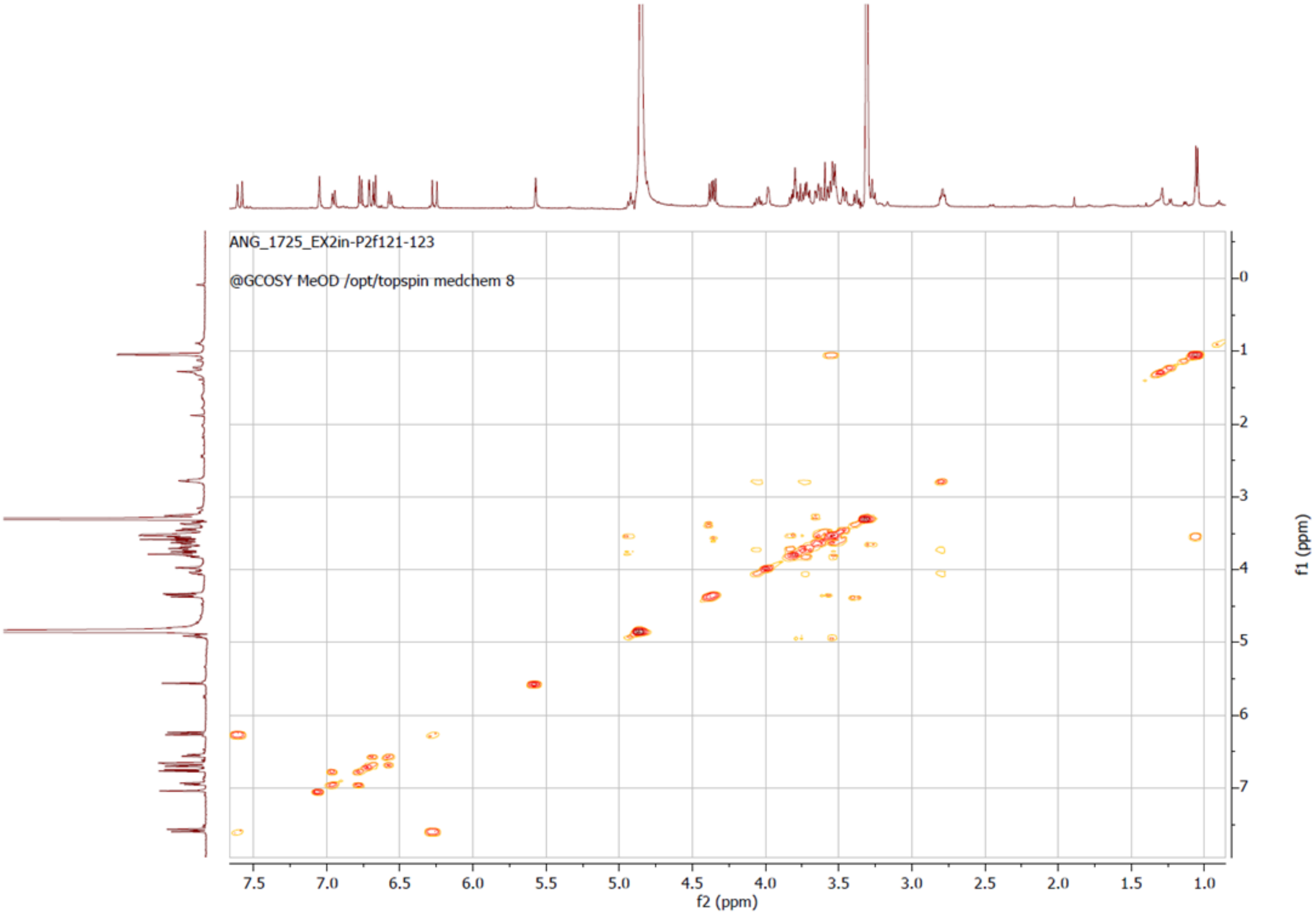

Figure S13. COSY spectrum of teupolioside (CD_3OD)

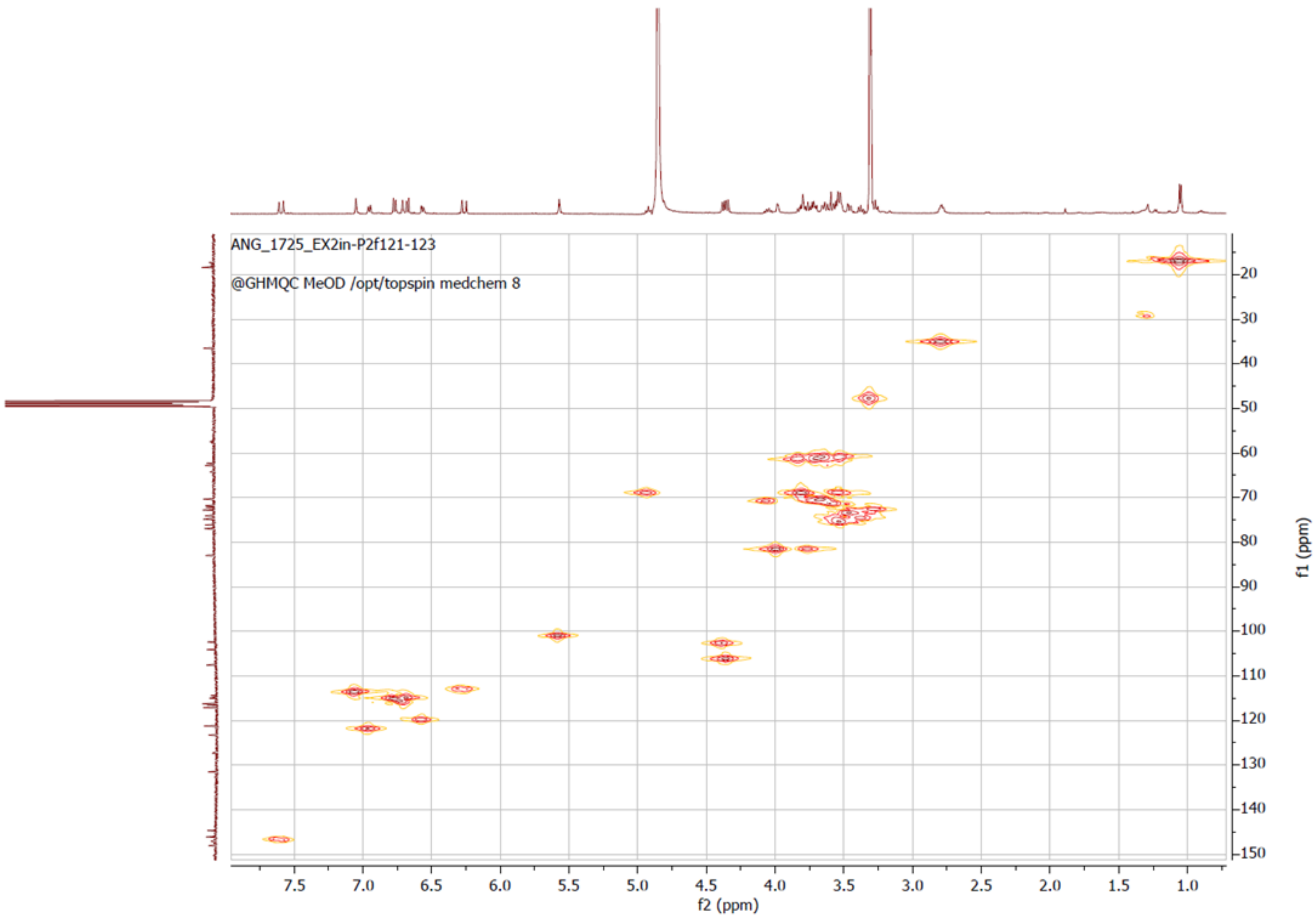

Figure S14. HSQC spectrum of teupolioside (CD_3OD)

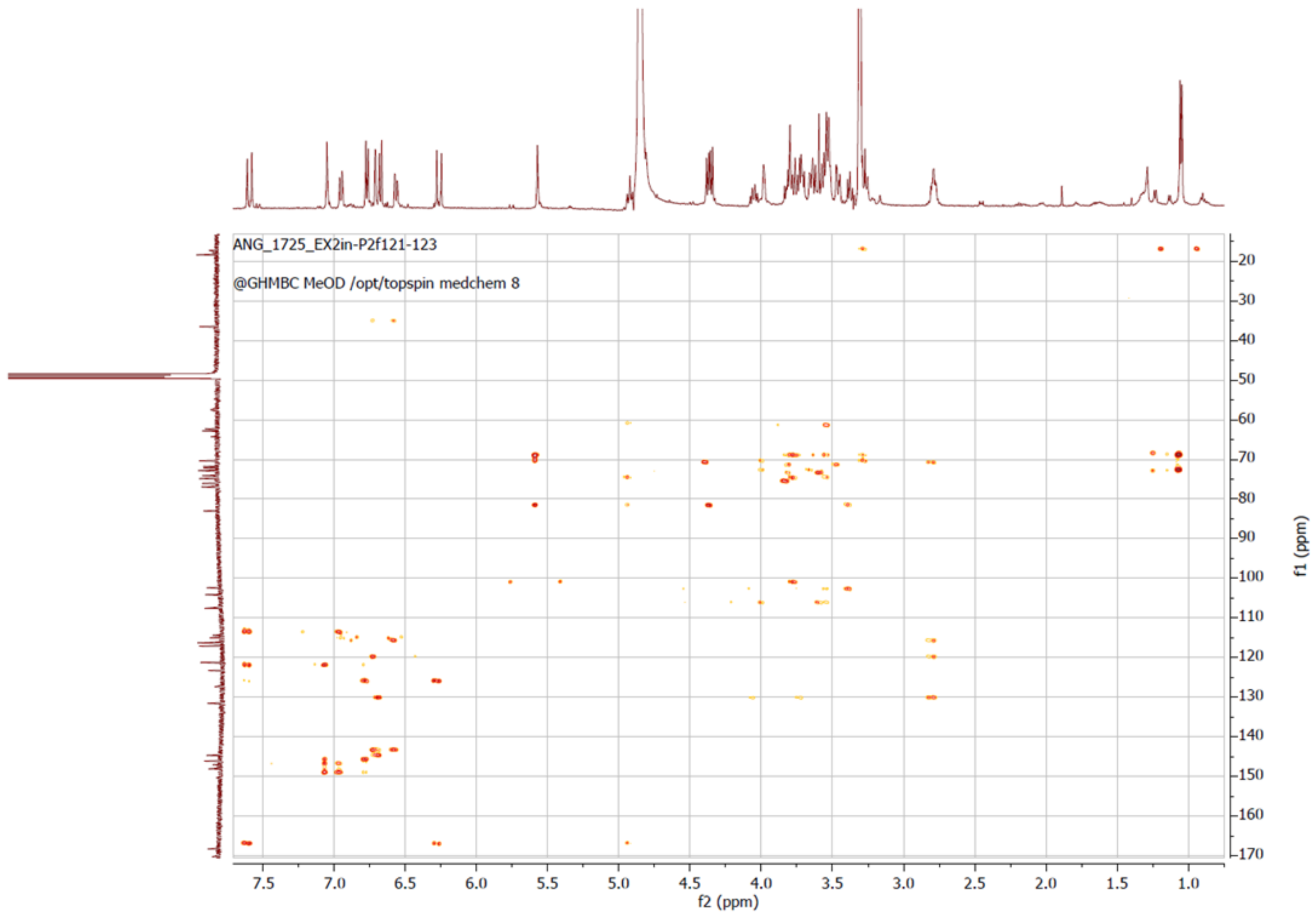

Figure S15. HMBC spectrum of teupolioside (CD_3OD)

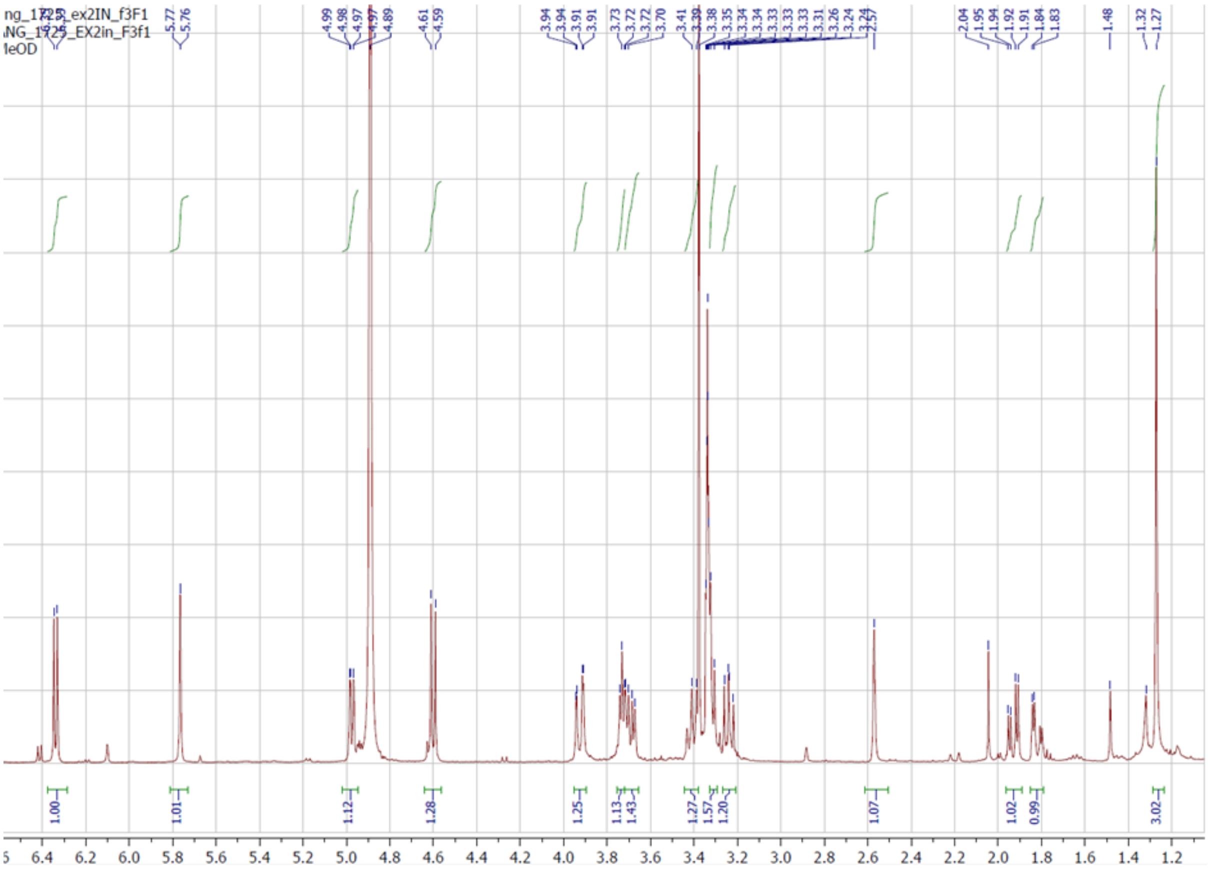

Figure S16. ^{1}H NMR spectrum of harpagide (CD_3OD, 400 MHz)

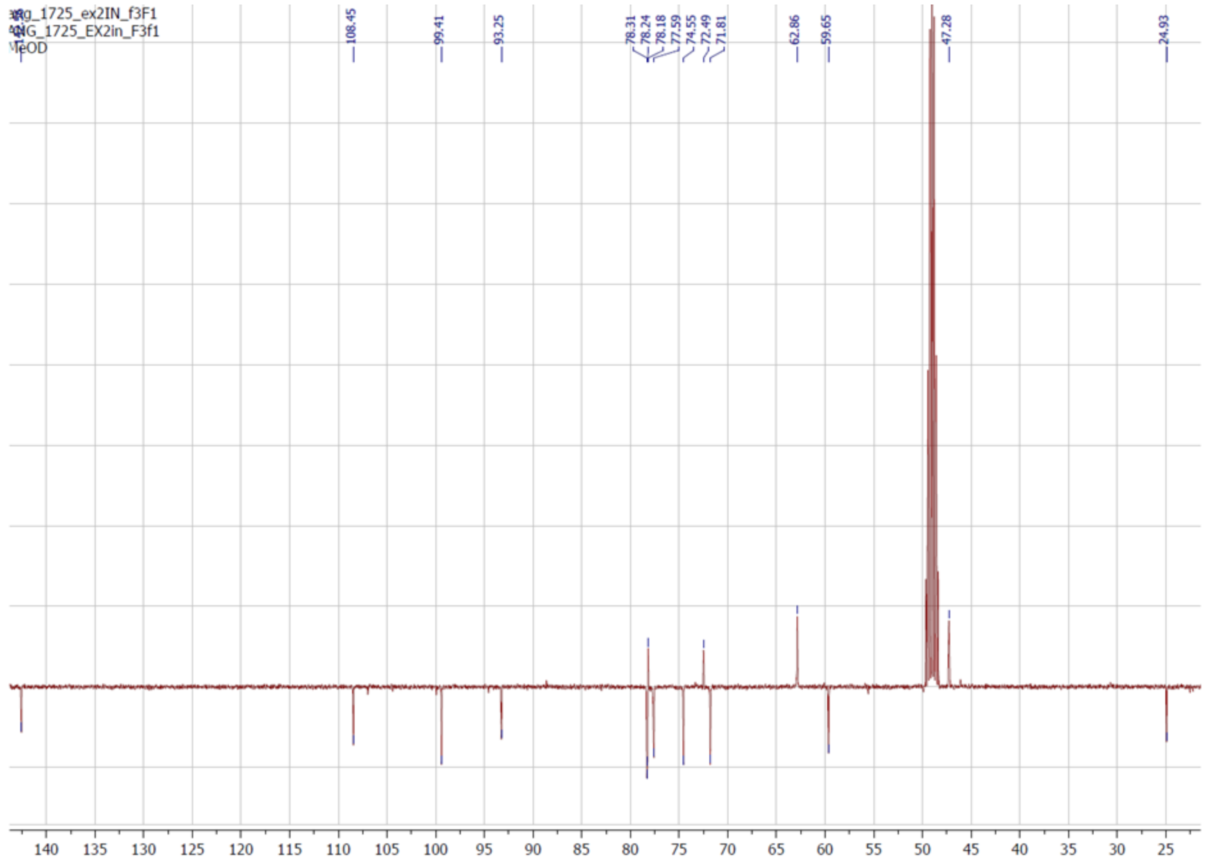

Figure S17. ^{13}C NMR spectrum of harpagide (CD_3OD, 100 MHz)

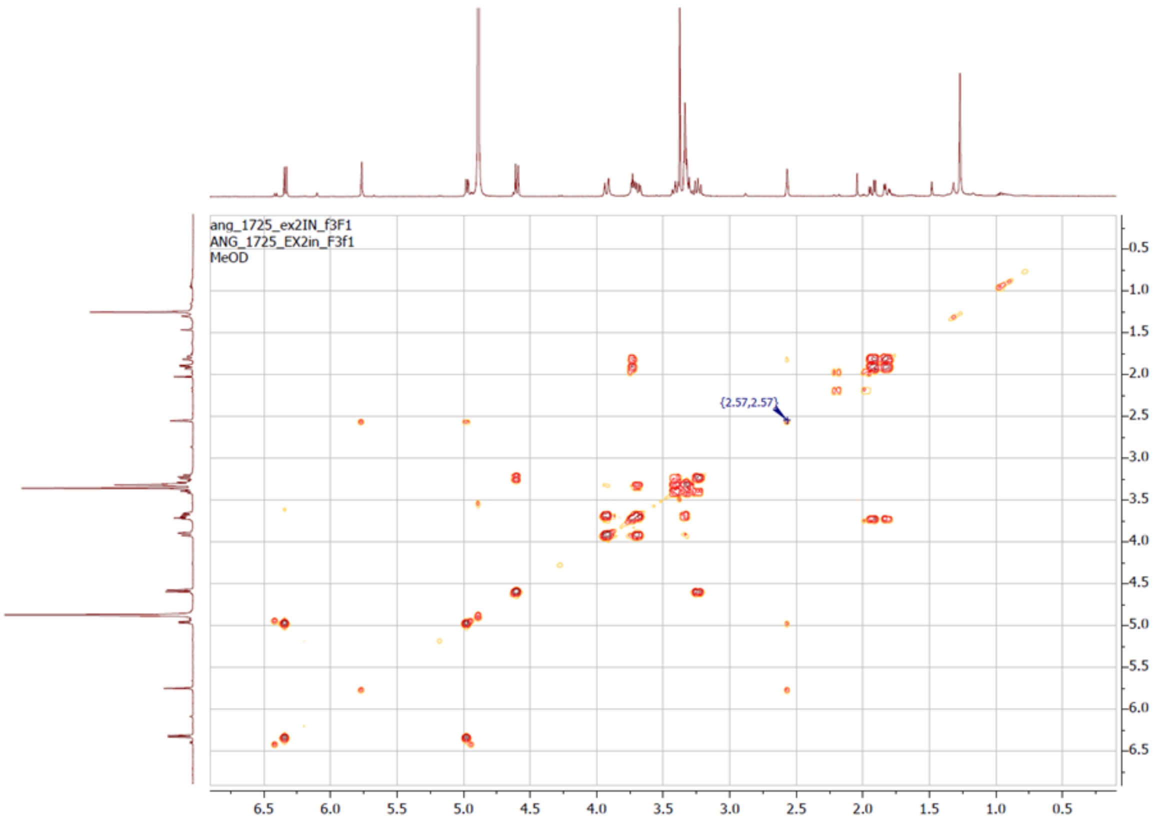

Figure S18. COSY spectrum of harpagide (CD_3OD)

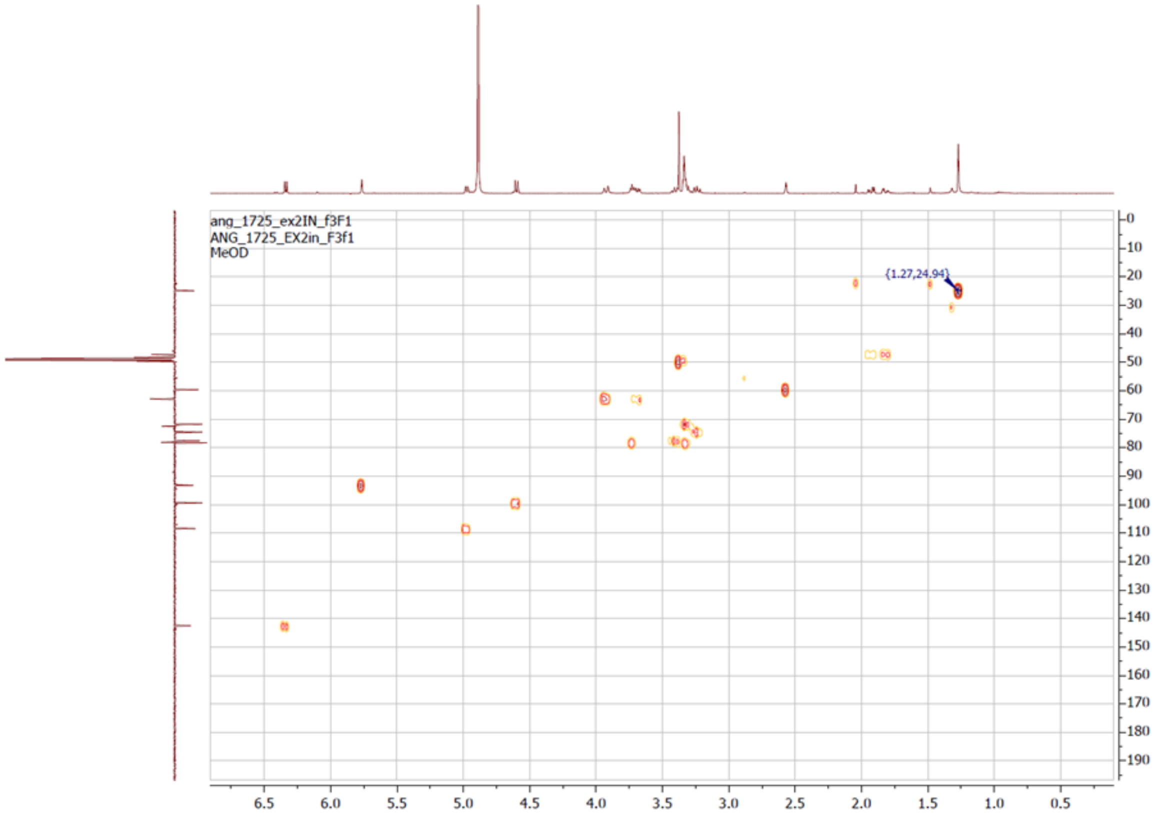

Figure S19. HSQC spectrum of harpagide (CD_3OD)

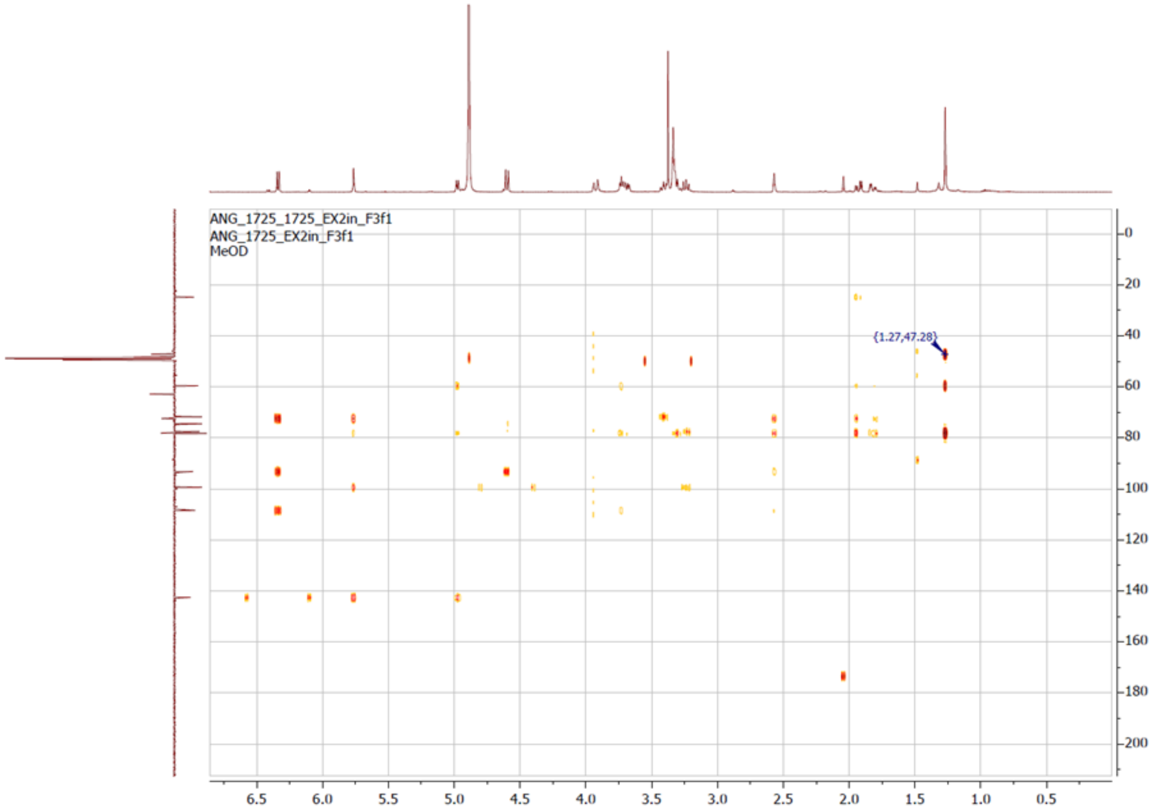

Figure S20. HMBC spectrum of harpagide (CD_3OD)

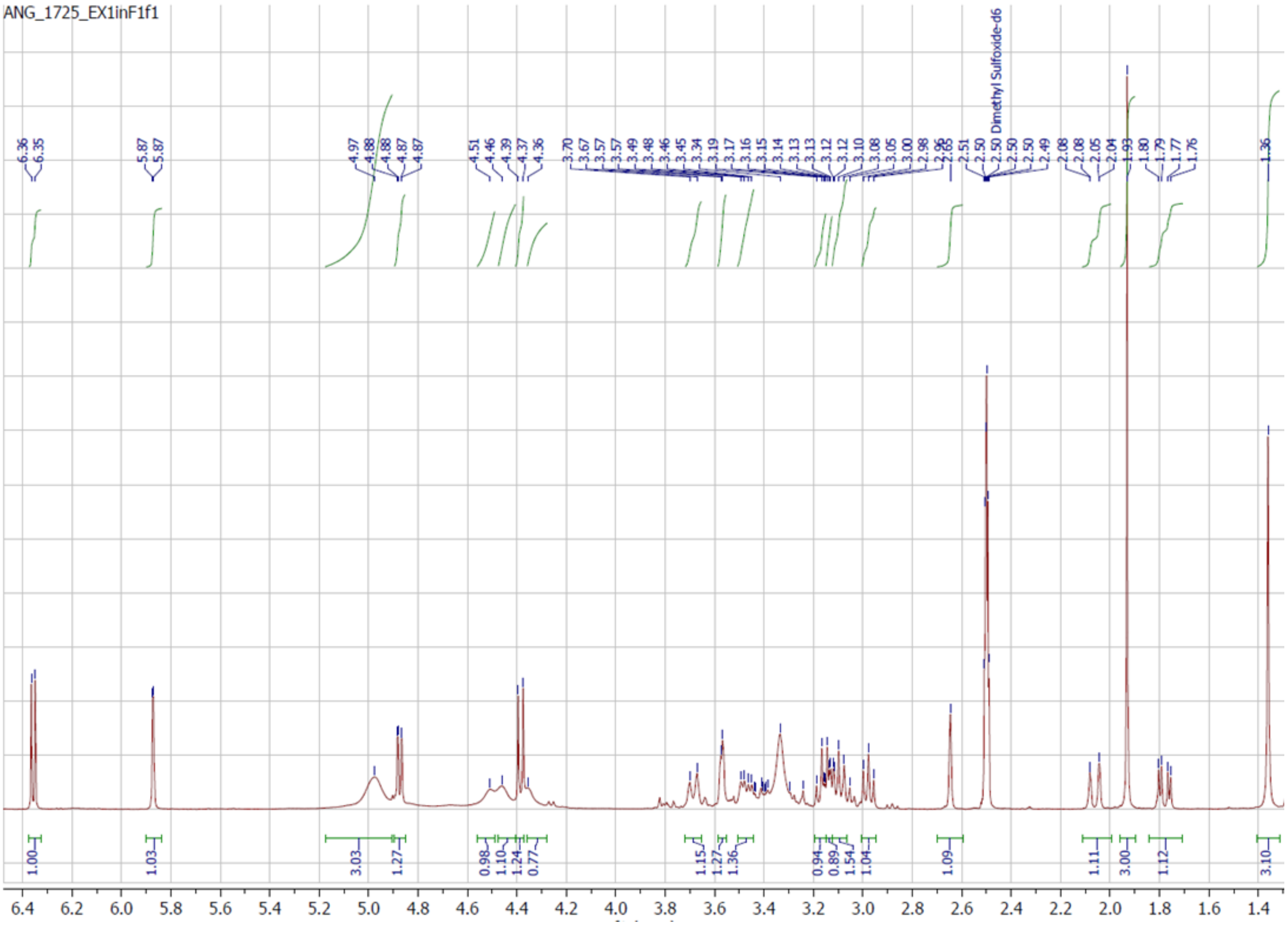

Figure S21. ^{1}H NMR spectrum of 8-*O*-acetylharpagide (DMSO-d_6, 400 MHz)

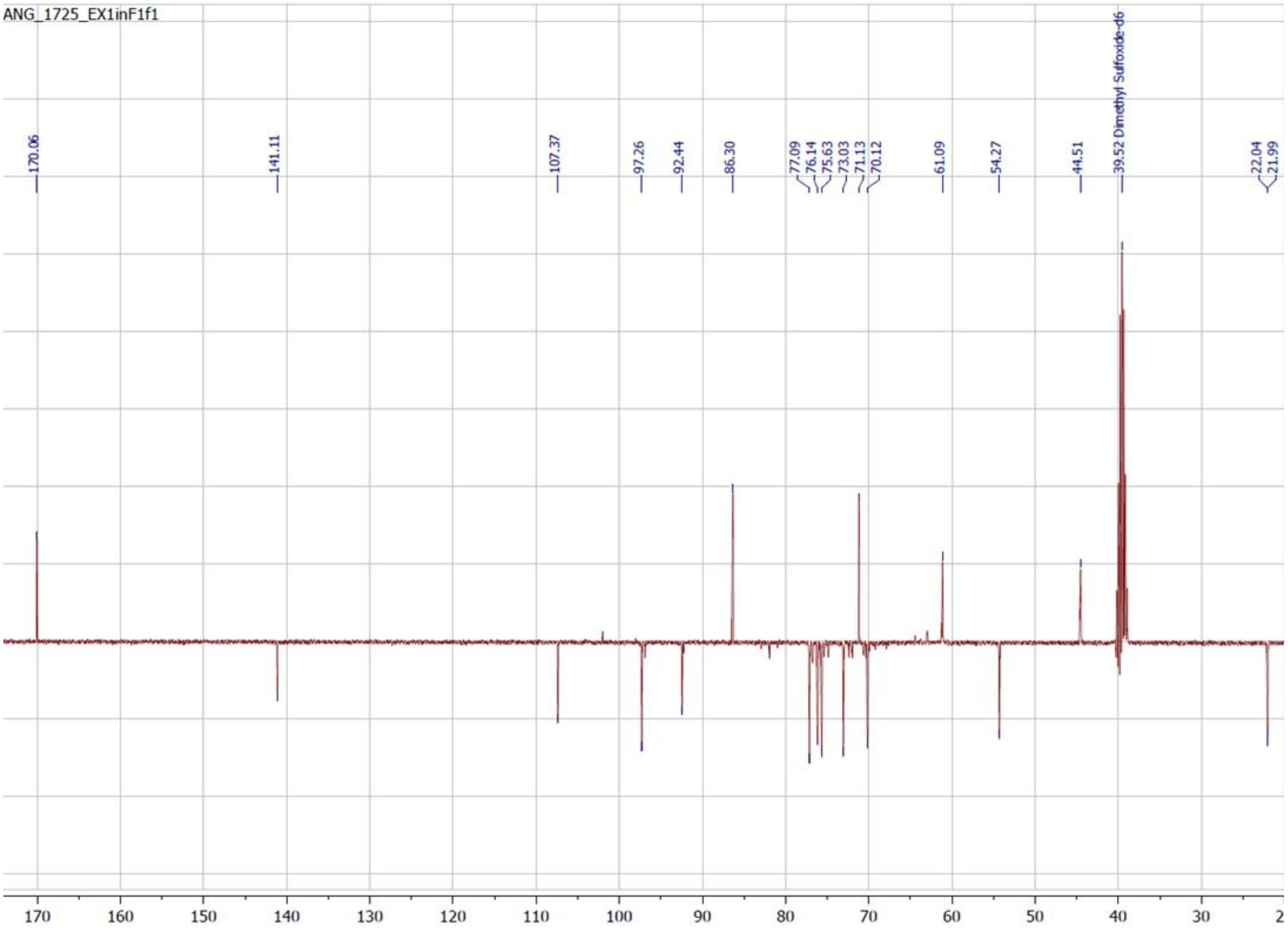

Figure S22. ^{13}C NMR spectrum of 8-*O*-acetylharpagide (DMSO-d_6, 100 MHz)

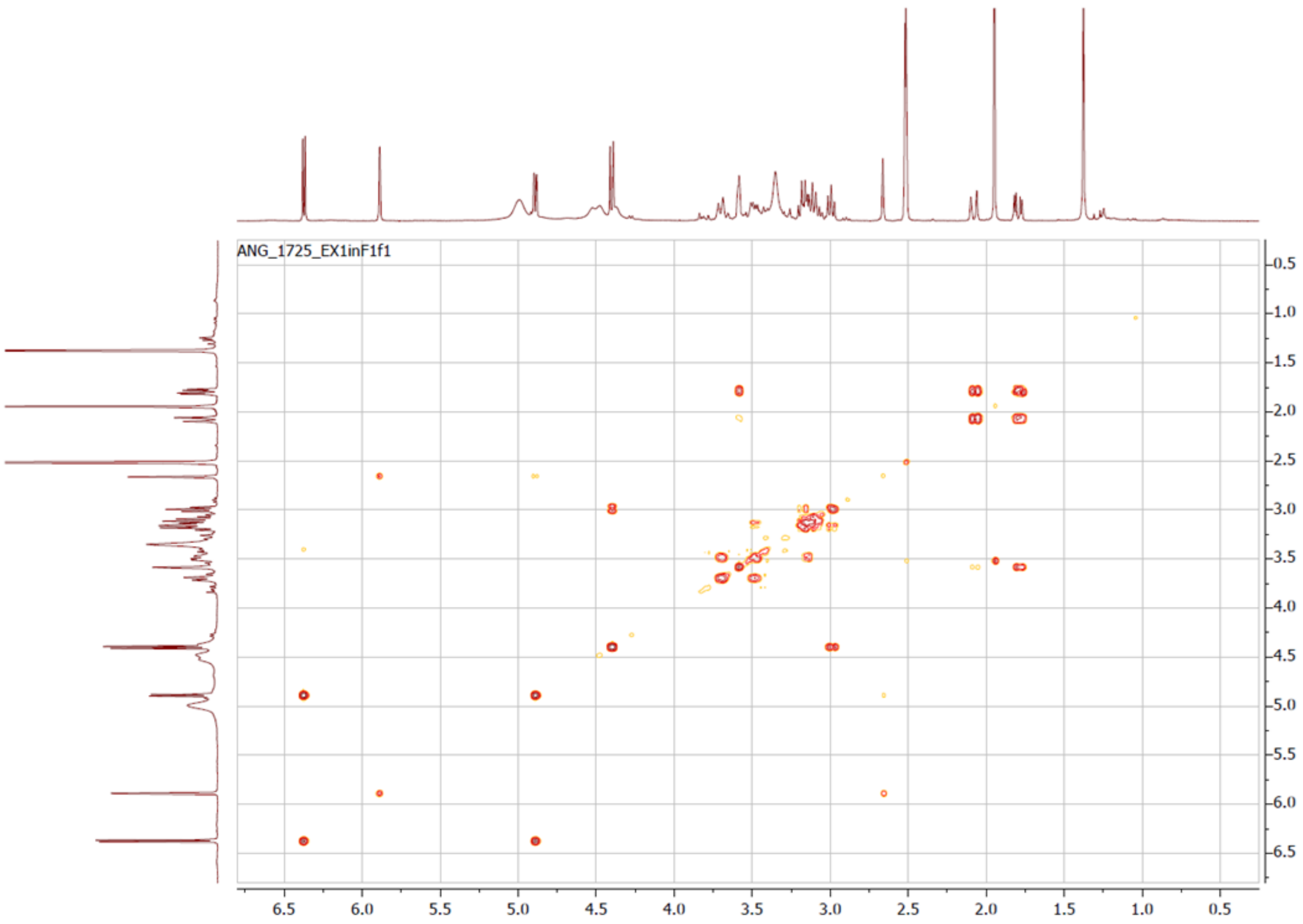

Figure S23. COSY spectrum of 8-*O*-acetylharpagide (DMSO-d_6)

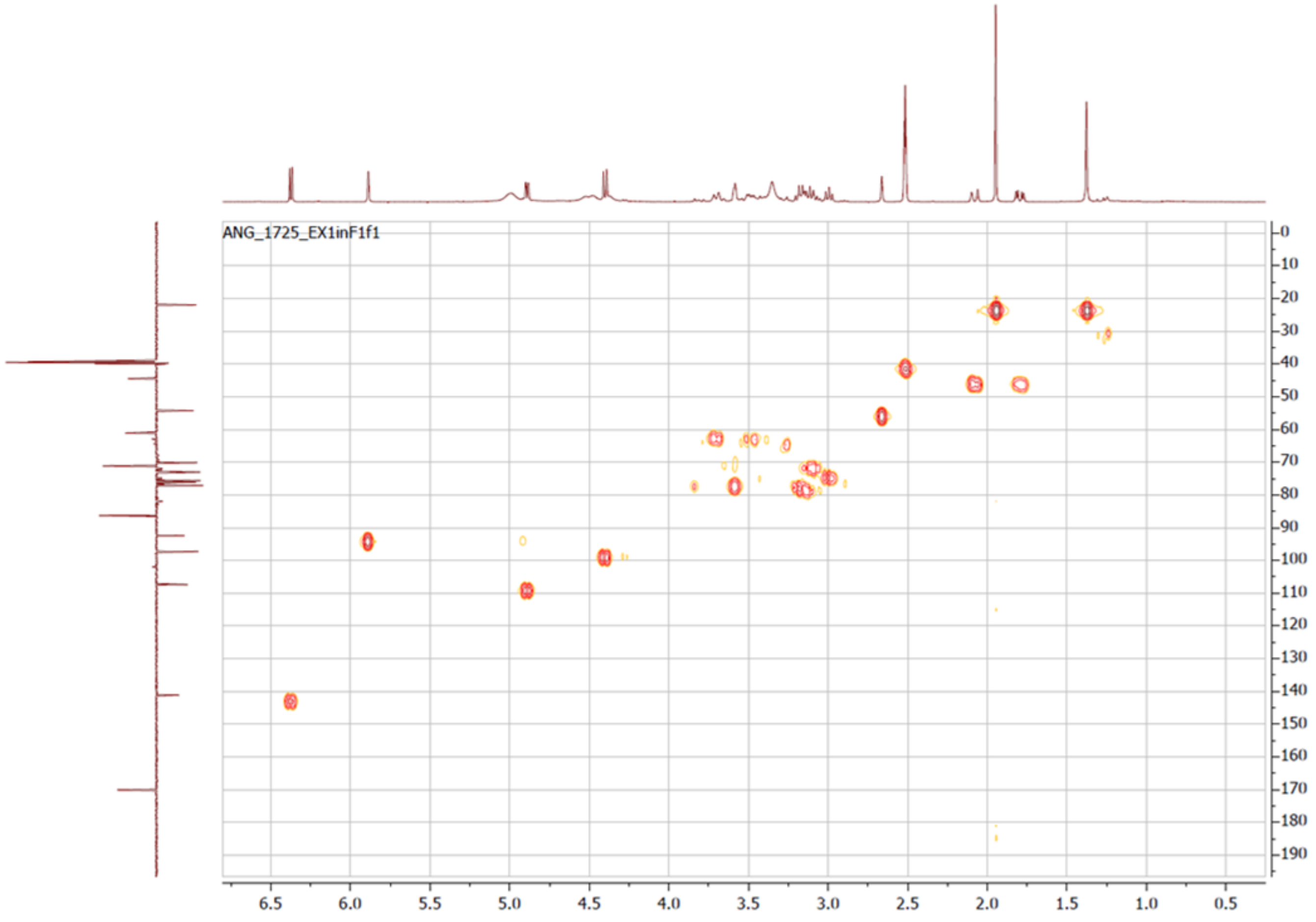

Figure S24. HSQC spectrum of 8-*O*-acetylharpagide (DMSO-d_6)

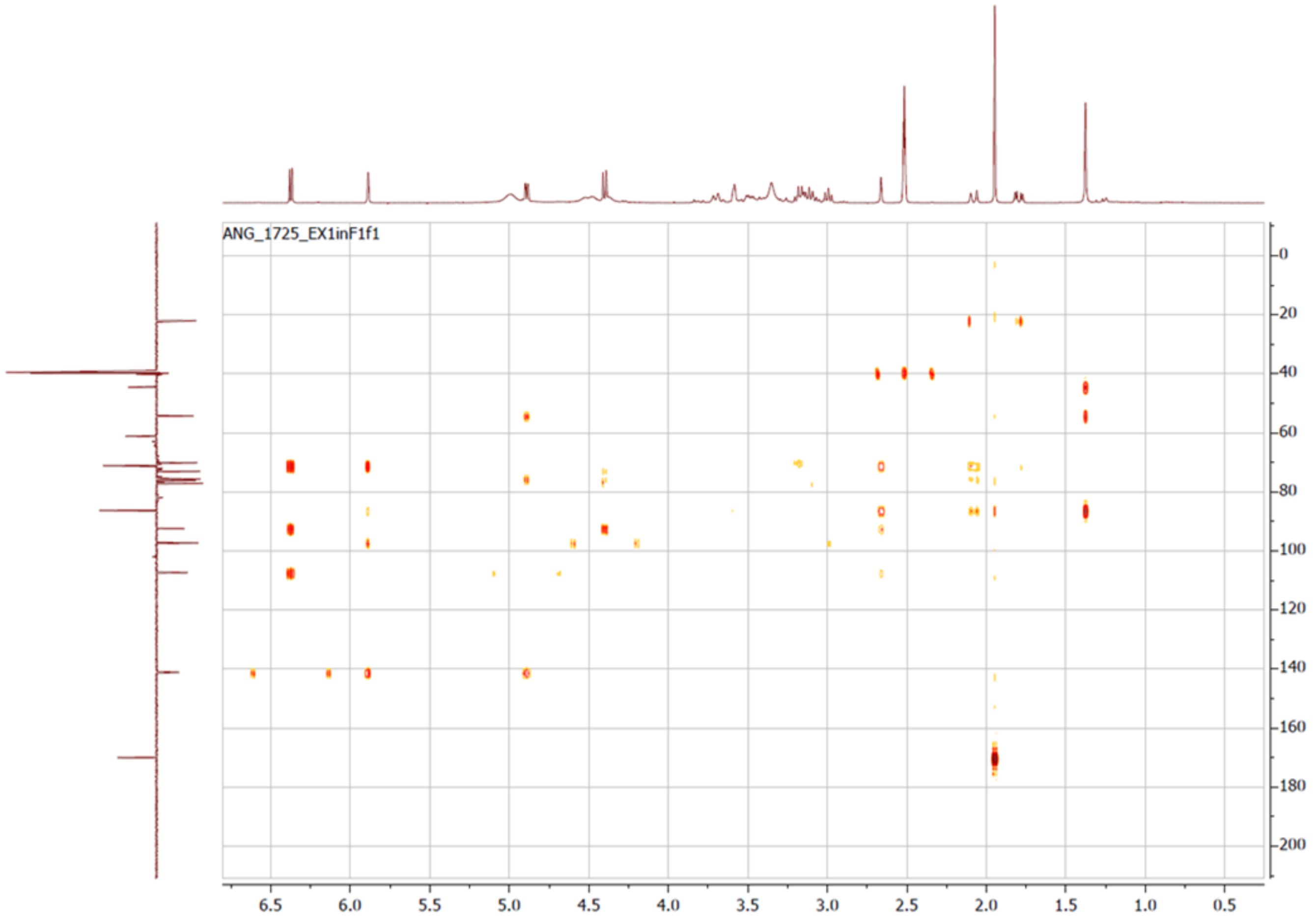

Figure S25. HMBC spectrum of 8-*O*-acetylharpagide (DMSO-d_6)

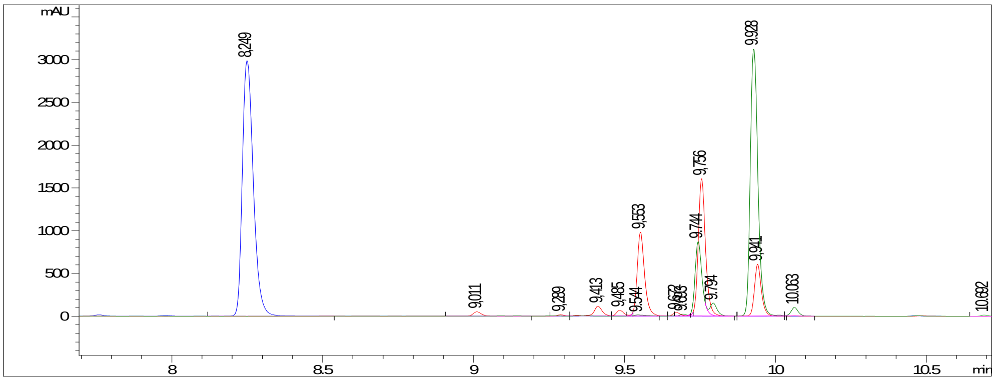

Figure S26. Acetylation reaction: HPLC chromatograms of harpagoside (blue), after 24 h of reaction (4 h at 70 °C and 20 h at room temperature, red) and after 6 more days of reaction (green)

SPL-0005 : Expression analysis of selected RNA

FD190914 - mQPA 43 genes	Genes		Control	Apolar extract		Intermediate extract		Polar extract		8-*O*-acetylharpagide		Polyphenols fraction		Iridoids fraction	
			-	2.74 x 10^{-4}%		0.0025%		0.06%		0.0024%		0.0024%		0.0226%	
			Cycles	Cycles	% control Mean HK	Cycles	% control Mean HK	Cycles	% control Mean HK	Cycles	% control Mean HK	Cycles	% control Mean HK	Cycles	% control Mean HK
	Abbreviation														
Housekeeping	GAPDH	Glyceraldehyde-3-phosphate dehydrogenase	19,23<br>19,13	18,98<br>18,84	134	18,97<br>19,08	125	18,84<br>18,78	144	19,07<br>18,96	110	18,88<br>18,85	126	18,86<br>18,80	161
	B2M	Beta-2-microglobulin	25,46<br>25,96	25,08<br>24,81	186	25,30<br>25,24	150	24,97<br>25,30	165	24,99<br>24,64	180	23,42<br>23,90	420	26,09<br>25,80	106
	RPLP0	Ribosomal protein, large, P0	19,30<br>19,24	19,68<br>19,63	85	19,54<br>19,46	96	19,84<br>19,68	80	19,18<br>19,19	104	19,25<br>19,50	95	20,00<br>19,99	76
	RPS28	Ribosomal protein S28	19,48<br>19,45	20,02<br>20,02	75	20,11<br>20,04	74	20,15<br>20,18	69	19,67<br>19,73	83	19,99<br>20,03	70	20,70<br>20,74	53
Keratinocytes differentiation	FLG	Filaggrin	27,08<br>27,26	27,49<br>27,59	86	27,14<br>27,13	115	26,63<br>26,53	168	26,52<br>26,34	163	26,66<br>26,65	145	27,65<br>27,72	88
	IVL	Involucrin	27,98<br>27,96	27,94<br>28,06	109	27,93<br>27,88	118	26,17<br>26,18	387	27,11<br>27,25	169	27,46<br>27,56	140	28,01<br>28,29	112
	KRT10	Keratin 10	24,52<br>24,37	24,28<br>24,47	116	24,54<br>24,46	108	22,79<br>22,84	345	24,12<br>23,88	133	23,54<br>23,81	174	24,51<br>24,63	116
	KRT5	keratin 5, type II	22,30<br>22,28	21,83<br>21,81	153	22,12<br>22,11	127	22,04<br>22,06	132	21,90<br>22,15	118	22,16<br>22,09	114	23,09<br>23,10	72
	LOR	Loricrin	26,94<br>26,91	27,24<br>27,60	79	27,11<br>27,22	95	27,71<br>27,68	65	26,16<br>26,64	143	26,87<br>26,84	107	27,50<br>27,51	84
	TGM1	transglutaminase 1	25,69<br>25,72	26,50<br>26,43	65	27,15<br>27,33	39	24,92<br>24,95	190	25,88<br>25,85	87	27,20<br>27,45	33	28,79<br>28,99	14
Extracellular matrix degradation	MMP1	matrix metallopeptidase 1	27,27<br>27,26	26,55<br>26,62	177	26,62<br>26,57	179	26,73<br>26,85	155	26,01<br>26,09	227	25,78<br>25,83	279	25,32<br>25,29	491
	MMP9	matrix metallopeptidase 9	32,18<br>32,69	31,83<br>32,00	157	32,22<br>31,89	145	31,79<br>31,65	181	31,25<br>31,70	189	30,85<br>31,01	284	31,79<br>31,43	222
	TIMP1	TIMP metallopeptidase inhibitor 1	21,80<br>21,68	22,25<br>22,06	83	22,02<br>21,91	96	22,44<br>22,44	69	21,98<br>21,90	85	21,83<br>21,90	93	20,88<br>20,90	227
	TIMP2	TIMP metallopeptidase inhibitor 2	31,13<br>31,04	30,04<br>29,91	239	30,25<br>30,28	198	30,18<br>30,42	193	30,03<br>30,57	171	30,27<br>30,08	191	30,58<br>30,74	170
Apoptosis	AKT1	V-akt murine thymoma viral oncogene homolog 1	28,22<br>28,70	27,50<br>27,31	227	27,50<br>27,61	208	27,05<br>27,04	294	26,94<br>27,04	267	26,67<br>26,80	332	27,48<br>27,78	222
	BAX	BCL2-associated X protein	28,29<br>28,32	28,06<br>27,93	137	28,20<br>28,12	124	27,92<br>27,92	146	27,66<br>27,77	147	27,69<br>27,85	147	28,69<br>29,09	85
	CASP3	Caspase 3, apoptosis-related cysteine peptidase	29,09<br>29,01	27,09<br>27,17	419	27,16<br>27,09	426	26,99<br>26,91	478	26,98<br>27,02	405	26,69<br>26,67	525	26,97<br>27,02	524
	FOS	FBJ murine osteosarcoma viral oncogene homolog	31,77<br>32,27	31,04<br>30,76	238	30,92<br>31,04	228	29,68<br>29,85	526	30,35<br>30,95	254	30,23<br>30,08	365	31,53<br>31,57	172
	TP53	Tumor protein p53	27,94<br>28,11	26,85<br>26,86	249	27,48<br>27,30	174	26,98<br>27,02	227	27,10<br>27,02	191	26,79<br>26,85	234	28,17<br>28,45	104
Cell-cell interactions	CDH1	cadherin 1, type 1	24,93<br>24,94	24,51<br>24,44	152	24,97<br>25,42	95	23,75<br>24,47	204	23,74<br>23,74	224	23,65<br>23,90	228	24,07<br>24,46	203
	CLDN1	claudin 1	24,27<br>24,10	23,30<br>23,25	208	24,09<br>24,03	122	22,92<br>23,08	254	22,78<br>23,06	236	23,71<br>23,93	131	25,49<br>25,54	50
	DSC1	desmocollin 1	32,18<br>32,31	30,73<br>30,86	302	31,25<br>30,86	258	28,00<br>28,06	2071	30,53<br>30,11	375	30,03<br>29,91	492	33,19<br>33,83	54
	DSP	desmoplakin	25,99<br>25,88	23,50<br>23,28	648	23,54<br>23,26	654	22,78<br>22,78	993	23,76<br>23,60	467	22,73<br>22,70	946	24,28<br>24,19	410
	ITGA6	Integrin, alpha 6	27,80<br>27,71	25,76<br>25,75	443	25,59<br>25,52	516	24,86<br>25,42	696	25,70<br>25,74	400	24,60<br>24,73	865	25,67<br>25,72	526
	ITGB1	integrin, beta 1 (fibronectin receptor, beta polypeptide, antigen CD29 includes MDF2, MSK12)	21,92<br>21,76	21,10<br>21,07	187	21,37<br>21,21	164	21,21<br>21,13	177	21,01<br>21,18	164	20,85<br>20,94	195	21,07<br>21,02	219
	LGALS1	Lectin, galactoside-binding, soluble, 1	21,94<br>21,92	22,33<br>22,32	84	22,47<br>22,46	78	23,81<br>23,86	30	22,30<br>22,62	68	22,30<br>22,47	74	23,59<br>23,60	40
	LGALS7	lectin, galactoside-binding, soluble, 7	23,86<br>23,93	25,44<br>25,39	39	25,47<br>25,58	36	25,20<br>25,21	45	24,97<br>25,16	44	25,17<br>25,42	39	28,41<br>28,68	5
Response to oxidative and et cellular stress	GPX1	glutathione peroxidase 1	22,79<br>22,68	22,70<br>22,67	115	22,66<br>22,54	123	23,45<br>23,43	68	22,59<br>22,72	103	22,55<br>22,69	110	22,86<br>22,79	119
	HIF1A	Hypoxia inducible factor 1, alpha subunit (basic helix-loop-helix transcription factor)	25,74<br>25,78	25,07<br>25,12	176	25,12<br>25,14	174	24,83<br>24,91	207	24,72<br>24,87	191	24,75<br>24,64	213	25,47<br>25,53	151
	HMOX1	Heme oxygenase (decycling) 1	28,11<br>28,13	27,91<br>28,02	123	27,83<br>27,83	137	28,06<br>28,27	108	26,68<br>27,26	221	27,59<br>27,76	139	27,76<br>27,96	151
	HSPB1	heat shock 27kDa protein 1	18,65<br>18,69	19,21<br>19,30	74	19,72<br>19,58	57	18,98<br>19,01	89	19,24<br>19,18	67	19,33<br>19,49	61	21,52<br>21,51	18
	NFKB1	Nuclear factor of kappa light polypeptide gene enhancer in B-cells 1	25,97<br>25,79	25,78<br>25,80	118	25,86<br>25,77	117	25,42<br>25,24	163	24,98<br>25,25	166	25,20<br>25,24	160	25,77<br>25,82	134
	NQO1	NAD(P)H dehydrogenase, quinone 1	29,88<br>29,63	28,68<br>28,59	240	27,83<br>27,62	458	27,68<br>27,17	568	27,63<br>27,79	402	27,17<br>27,07	629	27,94<br>28,00	433
	SOD1	Superoxide dismutase 1, soluble	23,97<br>23,71	23,54<br>23,46	140	23,50<br>23,23	156	23,36<br>23,20	164	23,32<br>23,35	138	22,88<br>23,03	187	23,63<br>23,60	147
Cytokines, Chemokines	IL1A	interleukin 1, alpha	26,07<br>25,55	23,94<br>23,73	429	24,00<br>23,68	436	23,91<br>23,63	454	24,21<br>24,03	311	23,33<br>23,45	535	23,63<br>23,37	618
	IL6	interleukin 6	nd<br>nd	nd<br>nd	-	nd<br>nd	-	nd<br>nd	-	nd<br>nd	-	nd<br>nd	-	nd<br>nd	-
Translation regulation	MAPK1	mitogen-activated protein kinase 1	26,96<br>26,97	25,96<br>26,02	218	26,05<br>26,19	202	25,73<br>25,83	254	25,69<br>25,84	225	25,70<br>25,79	237	26,04<br>26,12	233
	MAPK14	mitogen-activated protein kinase 14	28,65<br>28,52	27,34<br>27,47	251	27,44<br>27,52	241	27,10<br>27,04	319	26,99<br>27,45	255	26,90<br>26,99	316	27,66<br>27,98	216
Cellular cycle / Growth factor / Transcription factors	EGFR	epidermal growth factor receptor	25,15<br>24,92	24,69<br>24,51	149	24,61<br>24,53	155	25,00<br>24,88	119	24,13<br>24,54	160	24,48<br>24,44	151	25,00<br>24,92	133
	JUN	Jun proto-oncogene	29,23<br>29,56	28,77<br>29,03	156	28,91<br>29,00	151	28,72<br>29,15	154	27,94<br>27,65	296	28,09<br>28,46	221	28,70<br>28,94	187
	NOTCH1	Notch 1	30,15<br>30,31	28,77<br>28,98	284	29,34<br>29,28	212	29,18<br>29,02	244	29,14<br>29,22	202	28,99<br>29,48	205	30,53<br>31,05	87
	TGFB1	Transforming growth factor, beta 1	25,45<br>25,31	24,74<br>24,67	177	24,85<br>24,77	167	24,73<br>24,73	175	24,63<br>24,82	154	24,59<br>24,50	181	24,77<br>24,71	196
	TP63	Tumor protein p63	29,44<br>29,22	26,68<br>26,58	718	27,09<br>26,97	552	26,43<br>26,48	816	27,32<br>27,54	365	26,54<br>26,63	679	27,24<br>27,55	484
	FOXO1	Forkhead box O1	26,68<br>26,68	26,96<br>26,96	91	26,81<br>26,84	102	26,88<br>26,90	96	25,90<br>26,10	157	26,52<br>26,46	116	27,18<br>27,50	80

Stimulation (Arbitrary selection): % >	200	(cyan)
Inhibition (Arbitrary selection) : % <	50	(green)
Results to be interpreted with caution (high cycle count indicating low relative expression, close to detection limit) >	31	(grey)
Not detected or non-compliant melting curve	nd	

Figure S27. Expression analysis of selected RNA for keratinocytes (NHEK) from sample 1

SPL-0005 : Expression analysis of selected RNA

FD190914 - mQPA 43 genes	Genes		Control	Apolar extract		Intermediate extract		Polar extract		8-*O*-acetylharpagide		Polyphenols fraction		Iridoids fraction	
			-	2.74 x 10^{-4}%		0.0025%		0.06%		0.0024%		0.0024%		0.0226%	
			Cycles	Cycles	% control Mean HK	Cycles	% control Mean HK	Cycles	% control Mean HK	Cycles	% control Mean HK	Cycles	% control Mean HK	Cycles	% control Mean HK
	Abbreviation														
Housekeeping	GAPDH	Glyceraldehyde-3-phosphate dehydrogenase	17,92<br>18,00	18,10<br>17,98	99	17,80<br>17,74	103	18,01<br>17,97	109	18,33<br>18,08	99	17,94<br>17,85	102	18,04<br>17,93	119
	B2M	Beta-2-microglobulin	25,16<br>24,82	24,50<br>24,23	161	25,72<br>25,24	65	25,64<br>25,13	86	24,58<br>24,62	153	25,89<br>25,48	60	25,46<br>25,34	91
	RPLP0	Ribosomal protein, large, P0	18,96<br>18,86	18,88<br>18,80	110	18,73<br>18,75	102	19,14<br>19,09	97	18,99<br>18,94	113	18,84<br>18,80	103	19,44<br>19,34	87
	RPS28	Ribosomal protein S28	19,23<br>19,30	19,49<br>19,50	89	19,25<br>19,26	91	19,68<br>19,74	82	19,74<br>19,74	84	19,37<br>19,30	93	20,12<br>20,02	69
Keratinocytes differentiation	FLG	Filaggrin	26,74<br>26,89	26,58<br>26,71	118	26,87<br>26,78	90	26,73<br>26,88	112	27,09<br>27,09	97	27,00<br>26,78	92	27,29<br>27,50	81
	IVL	Involucrin	27,12<br>27,11	26,80<br>27,06	119	27,44<br>27,47	71	25,76<br>25,83	278	27,50<br>27,28	97	27,68<br>27,65	66	27,59<br>27,49	90
	KRT10	Keratin 10	23,67<br>23,79	23,94<br>24,05	87	24,14<br>24,36	63	23,21<br>23,18	161	24,36<br>24,35	76	23,90<br>23,98	84	24,26<br>24,22	85
	KRT5	keratin 5, type II	20,54<br>20,68	20,78<br>20,94	88	21,18<br>21,18	61	20,97<br>20,95	87	21,07<br>21,09	85	21,30<br>21,39	58	22,62<br>22,55	31
	LOR	Loricrin	27,01<br>27,08	26,57<br>26,70	139	26,91<br>26,85	101	27,20<br>27,52	90	27,08<br>27,19	110	26,93<br>26,96	104	27,19<br>27,21	109
	TGM1	transglutaminase 1	23,77<br>23,83	23,90<br>24,04	93	25,63<br>25,60	26	23,71<br>23,67	120	24,59<br>24,31	75	26,01<br>25,97	21	27,42<br>27,50	10
Extracellular matrix degradation	MMP1	matrix metallopeptidase 1	26,58<br>26,70	26,72<br>26,99	90	26,59<br>26,28	105	26,73<br>26,60	110	26,87<br>26,76	104	25,87<br>25,76	172	24,99<br>25,04	374
	MMP9	matrix metallopeptidase 9	30,56<br>30,56	30,43<br>30,51	111	30,28<br>30,80	93	29,89<br>29,80	183	30,06<br>29,83	180	29,74<br>29,50	187	28,62<br>28,62	465
	TIMP1	TIMP metallopeptidase inhibitor 1	20,63<br>20,56	20,95<br>20,84	85	21,44<br>21,28	53	21,23<br>21,14	74	21,08<br>21,11	83	21,01<br>20,92	75	19,75<br>19,61	229
	TIMP2	TIMP metallopeptidase inhibitor 2	29,10<br>29,31	29,21<br>29,34	99	29,64<br>29,43	72	29,83<br>29,63	77	29,55<br>29,64	89	29,50<br>29,80	72	29,61<br>29,79	86
Apoptosis	AKT1	V-akt murine thymoma viral oncogene homolog 1	26,58<br>26,82	26,78<br>26,75	100	26,83<br>26,85	82	26,97<br>27,00	91	26,93<br>26,99	98	26,98<br>26,80	85	26,76<br>26,87	112
	BAX	BCL2-associated X protein	27,77<br>28,00	27,81<br>27,75	112	27,73<br>27,72	101	27,76<br>27,58	129	27,81<br>27,73	127	27,51<br>27,74	116	28,09<br>28,63	88
	CASP3	Caspase 3, apoptosis-related cysteine peptidase	26,65<br>26,84	26,44<br>26,62	121	26,86<br>26,70	88	26,83<br>26,88	103	26,89<br>26,90	105	26,56<br>26,66	106	26,55<br>26,52	140
	FOS	FBJ murine osteosarcoma viral oncogene homolog	28,69<br>28,85	28,96<br>29,10	87	28,47<br>28,29	118	28,01<br>28,19	177	28,48<br>28,55	140	27,94<br>28,12	162	28,80<br>28,99	111
	TP53	Tumor protein p53	26,68<br>26,71	26,55<br>26,87	104	26,34<br>26,45	111	26,93<br>26,82	98	26,75<br>26,82	110	26,70<br>26,52	103	28,02<br>28,11	47
Cell-cell interactions	CDH1	cadherin 1, type 1	23,49<br>23,13	23,91<br>24,14	63	23,60<br>23,82	68	23,27<br>23,05	123	23,28<br>23,43	113	23,04<br>23,59	98	22,85<br>22,75	171
	CLDN1	claudin 1	23,11<br>23,21	23,31<br>23,30	94	23,78<br>23,74	60	23,27<br>23,21	105	23,23<br>23,23	112	24,04<br>23,92	55	25,48<br>25,44	25
	DSC1	desmocollin 1	29,68<br>30,13	29,94<br>30,20	92	30,46<br>30,68	56	28,61<br>28,73	259	30,62<br>30,65	70	29,84<br>30,54	81	32,14<br>32,06	26
	DSP	desmoplakin	22,82<br>22,68	22,63<br>22,20	133	23,60<br>23,28	56	22,65<br>22,48	127	22,73<br>22,64	123	23,51<br>23,34	61	23,75<br>23,72	61
	ITGA6	Integrin, alpha 6	24,69<br>24,69	24,67<br>24,87	99	24,89<br>24,72	84	24,80<br>24,82	103	24,83<br>24,70	111	24,77<br>24,56	99	24,78<br>24,66	119
	ITGB1	integrin, beta 1 (fibronectin receptor, beta polypeptide, antigen CD29 includes MDF2, MSK12)	20,66<br>20,63	20,72<br>20,63	102	20,79<br>20,63	87	20,88<br>20,89	94	21,03<br>20,96	92	20,56<br>20,49	106	20,60<br>20,53	128
	LGALS1	Lectin, galactoside-binding, soluble, 1	21,32<br>21,24	21,58<br>21,54	86	21,46<br>21,37	82	22,95<br>22,92	35	22,02<br>22,02	70	21,52<br>21,51	82	22,36<br>22,29	59
	LGALS7	lectin, galactoside-binding, soluble, 7	23,53<br>23,60	23,75<br>23,83	89	23,73<br>23,72	81	23,88<br>23,94	88	24,55<br>24,52	60	23,86<br>23,81	81	25,58<br>25,64	29
Response to oxidative and et cellular stress	GPX1	glutathione peroxidase 1	22,32<br>22,29	22,32<br>22,29	105	21,79<br>21,79	129	22,97<br>22,96	71	22,62<br>22,59	95	22,05<br>21,97	119	22,53<br>22,48	106
	HIF1A	Hypoxia inducible factor 1, alpha subunit (basic helix-loop-helix transcription factor)	24,69<br>24,86	24,62<br>24,67	114	24,84<br>24,83	87	25,05<br>25,02	93	25,05<br>25,03	97	24,91<br>24,94	87	25,10<br>25,14	95
	HMOX1	Heme oxygenase (decycling) 1	27,61<br>27,79	27,16<br>27,62	131	27,74<br>27,86	84	27,91<br>27,99	94	27,57<br>27,80	119	28,02<br>27,90	81	27,52<br>27,68	130
	HSPB1	heat shock 27kDa protein 1	18,64<br>18,71	18,97<br>18,93	86	20,01<br>20,00	36	19,12<br>19,13	82	19,53<br>19,53	65	19,99<br>20,00	39	21,24<br>21,27	20
	NFKB1	Nuclear factor of kappa light polypeptide gene enhancer in B-cells 1	25,30<br>25,51	25,18<br>25,23	120	25,50<br>25,47	85	25,29<br>25,36	118	25,48<br>25,32	118	25,37<br>25,38	99	25,32<br>25,31	129
	NQO1	NAD(P)H dehydrogenase, quinone 1	26,61<br>26,74	26,45<br>26,36	126	26,81<br>26,79	83	26,04<br>25,88	183	26,07<br>25,85	193	26,85<br>27,06	80	25,99<br>25,71	216
	SOD1	Superoxide dismutase 1, soluble	22,94<br>22,98	22,90<br>22,90	109	22,91<br>22,72	100	23,03<br>22,94	110	23,10<br>23,06	108	23,07<br>22,85	97	23,23<br>23,20	102
Cytokines, Chemokines	IL1A	interleukin 1, alpha	23,80<br>23,91	23,79<br>23,59	117	23,55<br>23,15	129	23,58<br>23,45	141	23,53<br>23,29	160	23,88<br>23,53	108	23,23<br>23,05	199
	IL6	interleukin 6	nd<br>nd	nd<br>nd	-	nd<br>nd	-	nd<br>nd	-	nd<br>nd	-	nd<br>nd	-	nd<br>nd	-
Translation regulation	MAPK1	mitogen-activated protein kinase 1	25,52<br>25,73	25,51<br>25,66	107	25,66<br>25,62	89	25,67<br>25,63	109	25,83<br>25,83	101	25,62<br>25,57	99	25,65<br>25,70	117
	MAPK14	mitogen-activated protein kinase 14	26,79<br>27,02	26,91<br>27,08	98	27,03<br>27,05	82	26,96<br>27,03	104	27,16<br>27,18	97	27,09<br>27,16	83	27,47<br>27,32	86
Cellular cycle / Growth factor / Transcription factors	EGFR	epidermal growth factor receptor	23,87<br>23,81	23,92<br>23,81	103	23,82<br>23,77	93	24,56<br>24,48	70	24,20<br>24,02	97	24,14<br>24,03	82	24,58<br>24,43	77
	JUN	Jun proto-oncogene	28,53<br>28,93	28,10<br>28,30	150	28,42<br>28,57	105	28,61<br>28,69	117	28,56<br>28,43	137	28,58<br>28,71	102	28,62<br>28,79	122
	NOTCH1	Notch 1	27,88<br>28,27	28,05<br>28,50	91	28,86<br>28,98	50	29,00<br>28,91	60	28,45<br>28,29	95	29,15<br>29,42	42	30,13<br>30,03	30
	TGFB1	Transforming growth factor, beta 1	23,86<br>23,93	23,98<br>23,92	101	23,95<br>23,91	88	24,13<br>23,98	100	24,10<br>24,06	103	24,09<br>24,09	85	23,87<br>23,75	129
	TP63	Tumor protein p63	26,02<br>26,14	26,04<br>26,22	101	26,20<br>26,27	81	26,58<br>26,43	83	26,50<br>26,49	88	26,32<br>26,25	84	26,89<br>26,89	69
	FOXO1	Forkhead box O1	26,56<br>26,66	26,21<br>26,28	135	26,71<br>26,81	81	26,93<br>26,86	91	26,68<br>26,76	109	26,88<br>26,98	78	27,10<br>27,14	85

Stimulation (Arbitrary selection): % >	200	(cyan)
Inhibition (Arbitrary selection) : % <	50	(green)
Results to be interpreted with caution (high cycle count indicating low relative expression, close to detection limit) >	31	(grey)
Not detected or non-compliant melting curve	nd	

Figure S28. Expression analysis of selected RNA for keratinocytes (NHEK) from sample 2

SPL-0005 : Expression analysis of selected RNA

FD190914 - mQPA 43 genes	Genes		Control	Apolar extract		Intermediate extract		Polar extract		8-*O*-acetylharpagide		Polyphenols fraction		Iridoids fraction	
			-	2.74 x 10^{-4}%		0.0025%		0.06%		0.0024%		0.0024%		0.0226%	
	Abbreviation		Cycles	Cycles	% control Mean HK	Cycles	% control Mean HK	Cycles	% control Mean HK	Cycles	% control Mean HK	Cycles	% control Mean HK	Cycles	% control Mean HK
Housekeeping	GAPDH	Glyceraldehyde-3-phosphate dehydrogenase	18,16<br>18,14	18,58<br>18,53	97	18,43<br>18,55	98	18,52<br>18,49	109	18,30<br>18,17	106	18,15<br>18,00	104	18,89<br>18,97	97
	B2M	Beta-2-microglobulin	25,59<br>25,58	26,09<br>25,65	106	26,80<br>26,50	60	24,99<br>25,09	203	25,60<br>25,84	103	25,80<br>25,87	83	25,89<br>26,05	128
	RPLP0	Ribosomal protein, large, P0	18,90<br>18,83	19,08<br>19,19	106	19,03<br>19,10	108	19,51<br>19,38	93	19,13<br>19,14	93	18,89<br>18,89	97	19,53<br>19,49	107
	RPS28	Ribosomal protein S28	19,36<br>19,38	19,69<br>19,80	99	19,78<br>19,83	92	20,00<br>20,03	89	19,62<br>19,59	96	19,48<br>19,37	95	20,18<br>20,17	96
Keratinocytes differentiation	FLG	Filaggrin	27,06<br>26,92	27,20<br>27,27	108	26,91<br>27,09	124	27,27<br>27,20	117	26,74<br>26,94	125	26,58<br>26,81	121	26,75<br>26,76	197
	IVL	Involucrin	27,67<br>27,70	27,76<br>27,73	123	27,75<br>27,67	122	26,53<br>26,53	309	27,35<br>27,44	137	27,33<br>27,21	132	27,18<br>27,13	242
	KRT10	Keratin 10	24,59<br>24,51	24,81<br>24,79	108	24,67<br>24,70	113	23,84<br>23,93	220	24,42<br>24,59	116	24,02<br>24,05	141	24,53<br>24,58	167
	KRT5	keratin 5, type II	21,51<br>21,53	22,02<br>22,03	90	22,17<br>22,05	83	22,36<br>22,31	79	22,11<br>21,95	79	21,94<br>21,85	76	23,84<br>23,88	33
	LOR	Loricrin	26,61<br>26,87	26,92<br>27,06	107	26,77<br>26,97	114	27,09<br>27,28	102	26,49<br>26,53	131	26,46<br>26,52	117	26,44<br>26,49	202
	TGM1	transglutaminase 1	24,93<br>24,85	25,51<br>25,51	83	26,47<br>26,49	41	25,29<br>25,23	107	24,99<br>25,09	101	27,09<br>27,03	22	28,11<br>28,03	18
Extracellular matrix degradation	MMP1	matrix metallopeptidase 1	26,00<br>26,01	25,98<br>26,11	125	25,12<br>25,08	233	25,51<br>25,54	194	26,08<br>26,04	108	24,99<br>25,03	197	24,96<br>24,98	343
	MMP9	matrix metallopeptidase 9	30,90<br>30,58	30,63<br>31,07	119	31,26<br>31,19	88	29,77<br>30,09	243	30,61<br>30,59	123	30,05<br>29,89	167	29,86<br>30,07	285
	TIMP1	TIMP metallopeptidase inhibitor 1	21,02<br>20,96	21,61<br>21,59	84	21,69<br>21,56	80	21,66<br>21,57	90	21,34<br>21,21	92	21,14<br>21,13	89	19,85<br>19,82	372
	TIMP2	TIMP metallopeptidase inhibitor 2	28,96<br>29,23	29,15<br>29,57	107	29,30<br>29,50	101	29,97<br>30,08	73	29,41<br>29,43	89	29,21<br>29,52	82	30,57<br>30,54	61
Apoptosis	AKT1	V-akt murine thymoma viral oncogene homolog 1	26,71<br>26,85	26,97<br>27,18	104	26,83<br>27,14	109	27,20<br>27,19	104	27,02<br>27,20	89	26,56<br>26,70	109	27,84<br>27,81	81
	BAX	BCL2-associated X protein	27,58<br>27,82	27,53<br>27,64	138	27,94<br>28,04	102	27,81<br>28,05	118	27,85<br>28,03	95	27,58<br>27,45	112	28,66<br>28,71	84
	CASP3	Caspase 3, apoptosis-related cysteine peptidase	26,85<br>26,87	26,80<br>27,04	123	26,78<br>26,90	126	26,82<br>26,83	142	27,00<br>27,23	94	26,68<br>26,51	119	27,49<br>27,30	116
	FOS	FBJ murine osteosarcoma viral oncogene homolog	28,10<br>28,50	28,45<br>28,59	109	28,10<br>28,47	126	28,22<br>27,99	158	28,03<br>28,19	127	27,82<br>27,88	134	29,82<br>29,60	63
	TP53	Tumor protein p53	26,33<br>26,61	26,78<br>26,99	96	26,97<br>27,03	86	26,75<br>26,89	109	26,74<br>26,83	90	26,55<br>26,59	92	29,04<br>28,93	29
Cell-cell interactions	CDH1	cadherin 1, type 1	23,43<br>23,89	23,01<br>23,03	197	22,51<br>23,21	220	22,94<br>22,83	235	23,01<br>23,74	140	23,10<br>22,52	179	23,21<br>23,79	188
	CLDN1	claudin 1	23,94<br>23,90	24,52<br>24,64	81	24,80<br>24,83	67	23,72<br>23,61	166	23,95<br>23,91	112	24,97<br>25,00	47	26,24<br>26,46	31
	DSC1	desmocollin 1	32,13<br>32,54	31,19<br>31,10	289	32,05<br>31,97	155	31,52<br>31,44	249	32,01<br>32,46	121	31,55<br>31,62	164	32,27<br>32,42	165
	DSP	desmoplakin	23,51<br>23,65	23,63<br>23,72	120	23,64<br>23,96	107	23,21<br>23,24	177	23,90<br>23,64	99	23,71<br>23,44	99	25,02<br>24,90	64
	ITGA6	Integrin, alpha 6	24,71<br>24,78	24,80<br>24,87	120	24,67<br>24,65	132	24,72<br>24,81	137	25,13<br>24,92	93	24,42<br>24,17	135	25,64<br>25,56	92
	ITGB1	integrin, beta 1 (fibronectin receptor, beta polypeptide, antigen CD29 includes MDF2, MSK12)	20,83<br>20,81	21,07<br>21,01	110	20,98<br>20,99	111	21,10<br>21,05	116	21,17<br>21,02	93	20,50<br>20,45	125	21,01<br>21,00	147
	LGALS1	Lectin, galactoside-binding, soluble, 1	20,25<br>20,28	20,65<br>20,62	99	20,60<br>20,60	99	21,54<br>21,51	58	20,75<br>20,74	81	20,10<br>20,10	111	21,14<br>21,08	93
	LGALS7	lectin, galactoside-binding, soluble, 7	24,03<br>24,09	24,79<br>24,91	74	25,05<br>25,17	60	25,23<br>25,25	61	24,84<br>24,79	67	24,50<br>24,49	73	26,30<br>26,49	33
Response to oxidative and et cellular stress	GPX1	glutathione peroxidase 1	22,19<br>22,13	22,61<br>22,51	97	22,28<br>22,23	117	22,80<br>22,69	93	22,50<br>22,44	91	21,96<br>21,92	115	22,78<br>22,79	108
	HIF1A	Hypoxia inducible factor 1, alpha subunit (basic helix-loop-helix transcription factor)	24,81<br>24,91	24,91<br>25,05	118	24,91<br>24,97	118	24,91<br>24,98	131	24,93<br>24,94	107	24,68<br>24,72	110	25,09<br>25,21	137
	HMOX1	Heme oxygenase (decycling) 1	27,62<br>28,08	27,93<br>28,07	114	27,90<br>28,01	114	28,16<br>28,02	116	27,57<br>27,68	130	27,66<br>27,65	112	27,48<br>27,44	216
	HSPB1	heat shock 27kDa protein 1	19,74<br>19,83	20,34<br>20,37	86	20,98<br>21,04	53	20,38<br>20,38	92	20,30<br>20,33	78	20,92<br>20,97	44	22,34<br>22,31	29
	NFKB1	Nuclear factor of kappa light polypeptide gene enhancer in B-cells 1	25,26<br>25,30	25,58<br>25,56	105	25,51<br>25,46	108	24,68<br>24,66	212	25,55<br>25,48	96	25,05<br>25,01	117	25,29<br>25,28	167
	NQO1	NAD(P)H dehydrogenase, quinone 1	27,61<br>27,44	27,18<br>27,49	147	26,69<br>26,83	212	26,32<br>26,49	302	27,01<br>26,96	163	27,09<br>26,88	144	25,99<br>25,89	501
	SOD1	Superoxide dismutase 1, soluble	23,14<br>23,19	23,53<br>23,47	101	23,21<br>23,19	122	23,65<br>23,55	103	23,54<br>23,31	94	23,02<br>22,96	111	23,64<br>23,64	120
Cytokines, Chemokines	IL1A	interleukin 1, alpha	23,70<br>23,66	23,88<br>23,83	113	23,36<br>23,28	160	22,32<br>22,10	386	23,88<br>23,64	107	23,21<br>23,05	145	23,65<br>23,62	173
	IL6	interleukin 6	nd<br>nd	nd<br>nd	-	nd<br>nd	-	nd<br>nd	-	nd<br>nd	-	nd<br>nd	-	nd<br>nd	-
Translation regulation	MAPK1	mitogen-activated protein kinase 1	25,57<br>25,80	25,90<br>25,86	111	25,92<br>26,10	99	25,90<br>25,80	123	25,89<br>25,95	95	25,58<br>25,58	106	26,23<br>26,44	107
	MAPK14	mitogen-activated protein kinase 14	27,21<br>27,28	27,21<br>27,59	116	27,33<br>27,51	110	27,15<br>27,15	148	27,56<br>27,28	100	26,90<br>27,00	121	27,81<br>27,99	106
Cellular cycle / Growth factor / Transcription factors	EGFR	epidermal growth factor receptor	24,04<br>24,01	24,42<br>24,24	104	24,25<br>24,23	107	24,25<br>24,06	127	24,67<br>24,53	76	24,11<br>24,04	95	24,84<br>24,85	95
	JUN	Jun proto-oncogene	28,63<br>28,83	28,55<br>28,56	144	28,52<br>28,68	136	28,76<br>28,91	129	28,50<br>28,59	128	27,82<br>28,09	169	27,85<br>28,17	277
	NOTCH1	Notch 1	28,78<br>28,69	28,89<br>29,01	110	29,01<br>29,26	95	29,02<br>29,10	111	29,02<br>29,08	90	29,49<br>29,57	57	30,99<br>31,52	30
	TGFB1	Transforming growth factor, beta 1	24,07<br>24,08	24,50<br>24,30	102	24,22<br>24,17	115	24,46<br>24,28	113	24,35<br>24,29	95	23,95<br>23,98	106	24,83<br>24,78	101
	TP63	Tumor protein p63	26,31<br>26,57	26,78<br>26,86	98	26,75<br>26,81	98	25,94<br>25,98	193	26,66<br>26,77	93	26,57<br>26,59	89	28,29<br>28,29	46
	FOXO1	Forkhead box O1	26,74<br>26,83	26,77<br>26,89	124	26,84<br>26,84	120	26,83<br>26,82	135	26,64<br>26,80	118	26,58<br>26,52	116	26,58<br>26,53	196

Stimulation (Arbitrary selection): % >	200	
Inhibition (Arbitrary selection) : % <	50	
Results to be interpreted with caution (high cycle count indicating low relative expression, close to detection limit) >	31	
Not detected or non-compliant melting curve	nd	

Figure S29. Expression analysis of selected RNA for keratinocytes (NHEK) from sample 3

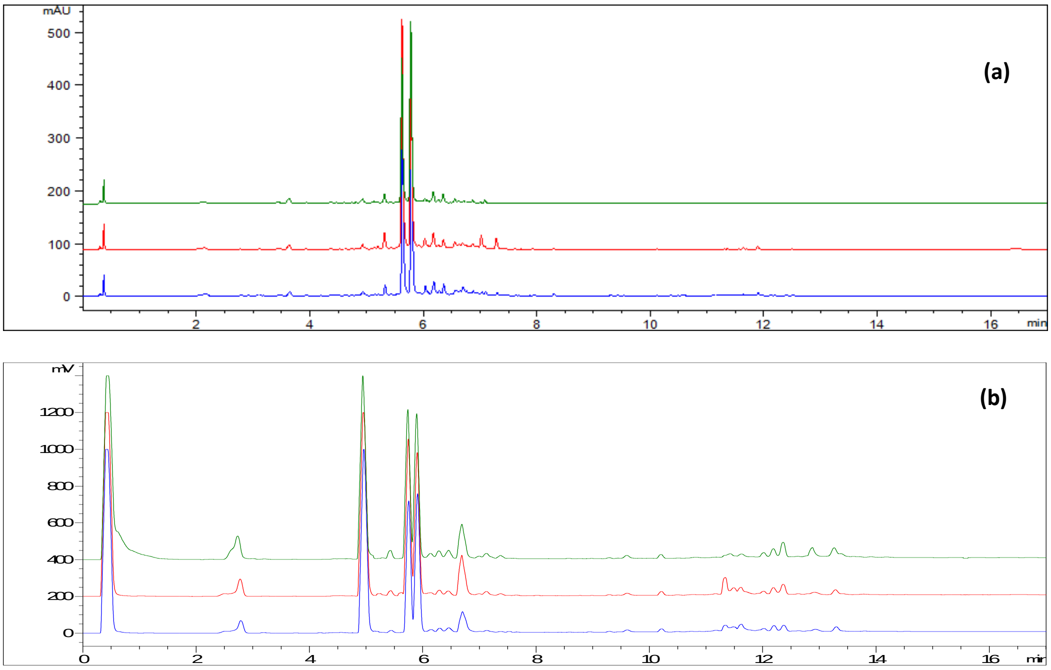

Figure S30. Interannual variability study on *Ajuga pyramidalis* ethanolic extracts, samples collected in 2017 (blue), 2018 (red), 2019 (green): **(a)** HPLC-DAD and **(b)** HPLC-ELSD chromatograms.
